# The HTLV-1 gp21 fusion peptide inhibits antigen specific T-cell activation *in-vitro* and in mice

**DOI:** 10.1371/journal.ppat.1007044

**Published:** 2018-05-04

**Authors:** Etai Rotem, Omri Faingold, Meital Charni, Yoel A. Klug, Daniel Harari, Liraz Shmuel-Galia, Alon Nudelman, Varda Rotter, Yechiel Shai

**Affiliations:** 1 Department of Biomolecular Sciences, The Weizmann Institute of Science, Rehovot, Israel; 2 Department of molecular cell biology, The Weizmann Institute of Science, Rehovot, Israel; University of Illinois at Chicago College of Medicine, UNITED STATES

## Abstract

The ability of the Lentivirus HIV-1 to inhibit T-cell activation by its gp41 fusion protein is well documented, yet limited data exists regarding other viral fusion proteins. HIV-1 utilizes membrane binding region of gp41 to inhibit T-cell receptor (TCR) complex activation. Here we examined whether this T-cell suppression strategy is unique to the HIV-1 gp41. We focused on T-cell modulation by the gp21 fusion peptide (FP) of the Human T-lymphotropic Virus 1 (HTLV-1), a Deltaretrovirus that like HIV infects CD4^+^ T-cells. Using mouse and human *in-vitro* T-cell models together with *in-vivo* T-cell hyper activation mouse model, we reveal that HTLV-1’s FP inhibits T-cell activation and unlike the HIV FP, bypasses the TCR complex. HTLV FP inhibition induces a decrease in Th1 and an elevation in Th2 responses observed in mRNA, cytokine and transcription factor profiles. Administration of the HTLV FP in a T-cell hyper activation mouse model of multiple sclerosis alleviated symptoms and delayed disease onset. We further pinpointed the modulatory region within HTLV-1’s FP to the same region previously identified as the HIV-1 FP active region, suggesting that through convergent evolution both viruses have obtained the ability to modulate T-cells using the same region of their fusion protein. Overall, our findings suggest that fusion protein based T-cell modulation may be a common viral trait.

## Author summary

In order to successfully infect and persist in their hosts, viruses utilize multiple strategies to evade the immune system. HIV utilizes membrane interacting regions of its envelope protein, primarily used to fuse with its target cells, to inhibit T-cell activation. Yet, it is unknown whether this ability is shared with other viruses. In this study we examined the T-cell inhibitory activity of the envelope protein of the Human T-lymphotropic virus 1 (HTLV-1), which infects T-cells. We focused on a functionally conserved region of HTLV’s and HIV’s fusion proteins, the fusion peptide (FP). Here, we reveal that HTLV’s FP inhibits the activity of T-cells *in-vitro* and in a T-cell hyper activation model in mice. This inhibition is characterized by downregulation of the T-cell Th1/type 1 response, leading to an elevated T-cell Th2/type 2 response observed by transition in the profiles of mRNA, cytokines and regulatory proteins. Furthermore, we demonstrate that the HTLV and HIV FPs inhibit T-cell activation at different levels of the signaling cascade. Although the HTLV FP’s mechanism of T-cell inhibition differs from the HIV’s FP, our findings suggest that FP mediated immune evasion might be a trait shared between different viruses.

## Introduction

The mutual evolutionary pressure between viruses and their hosts has driven viruses to adopt various immune evasion mechanisms [1–4]. Many evasion strategies of enveloped viruses, such as antigen presentation antagonism and glycan shielding, can be mediated by their fusion glycoproteins (reviewed in [5]). One of the most studied glycoproteins in this aspect is HIV’s gp41, which aside from its crucial role in virus-cell membrane fusion [6, 7], was shown to inhibit T-cell activity. This was proposed to occur during the fusion process using several membrane interacting segments [8–10], including the fusion peptide (FP) [11, 12] (reviewed in [9]). This strategy of modulating the immune response during membrane fusion has only been reported for HIV, although other enveloped viruses infect T-cells through membrane fusion as well [13–16]. We hypothesized that other human enveloped viruses might share HIV’s strategy of immune suppression.

To this aim we examined the immune modulatory ability of the human T-lymphotropic virus-1 (HTLV-1), which exploits CD4^+^ T-cells as its primary target cell population [17]. As both HTLV-1 and HIV-1 are members of the *retroviridae* family they share a common ancestor and similar genomic architecture [18, 19]. Their envelope proteins are similarly structured and are composed of two non-covalently bound subunits, gp46/gp21 in HTLV and gp120/gp41 in HIV, which bind cellular receptors and initiate fusion, respectively [20, 21]. Both viruses utilize several proteins to interfere with T-cell activity and manipulate the anti-viral immune response (23–25). HTLV’s p12 and p8 promote the proteosomal degradation of MHC-I and downregulate TCR complex signaling, respectively [22] while HIV’s Nef and Vpu downregulate MHC-I from the cell surface and promote internalization and degradation of CD4 in infected cells [23, 24]. Additionally, HTLV-1 has been previously reported to harbor an immunosuppressive domain (ISD) within its envelope transmembrane subunit gp21 that is conserved between different retroviral envelope proteins [25]. The ISD that is concealed by the envelope’s surface subunit [26, 27], has been reported to inhibit T-cell proliferation [25], to be crucial for viral infection *in vivo* [27] and to support tumor cells immune escape [26, 28, 29].

Suppression of TCR induced activation by HIV is well characterized and was shown to occur by targeting several TCR complex components via gp41 in the membrane [8, 9, 11, 30]. A membranotropic region of HTLV-1 gp21 is the FP that is concealed within the envelope complex. Following binding of the surface subunit to the cellular receptor, a conformational change exposes the FP leading to its insertion into the plasma membrane and to fusion with the host cell [31, 32]. Therefore, we decided to focus on the FP region as a possible immune suppressor of TCR activation in the membrane.

In this study we utilized *in-vitro* and *in-vivo* assays including T-cell proliferation and an experimental autoimmune encephalomyelitis (EAE) mouse model to investigate the ability of the HTLV-1 gp21 FP to interfere with T-cell activity. We reveal that the HTLV FP inhibits T-cell activation downstream of the TCR complex in contrast to the HIV FP that specifically targets the TCRα subunit. Moreover, the HTLV FP markedly reduced manifestation of an *in-vivo* EAE mouse model. Downregulation of T-cell activity was associated with reduced expression and secretion of Th1-specific cytokines and an elevated expression and secretion of Th2-specific cytokines. This transition in cytokine pattern was correlated to a decreased expression of T-bet and an elevated expression of Gata3, Th1- and Th2- specific transcription factors respectively. Interestingly, the HIV FP had no effect on both T-bet and Gata3 expression levels. This study suggests that in addition to its role in fusion, the HTLV FP interferes with T-cell activation by downregulating the type 1 anti-viral immune response, consequently leading to an elevated type 2 response. Overall, these findings reveal that like HIV, HTLV-1 adopted a similar strategy of immune suppression by it fusion protein, pointing to a possible prevailing trait of human T-cell viruses.

## Results

### The HTLV FP specifically inhibits T-cell activation

**Fig 1:**
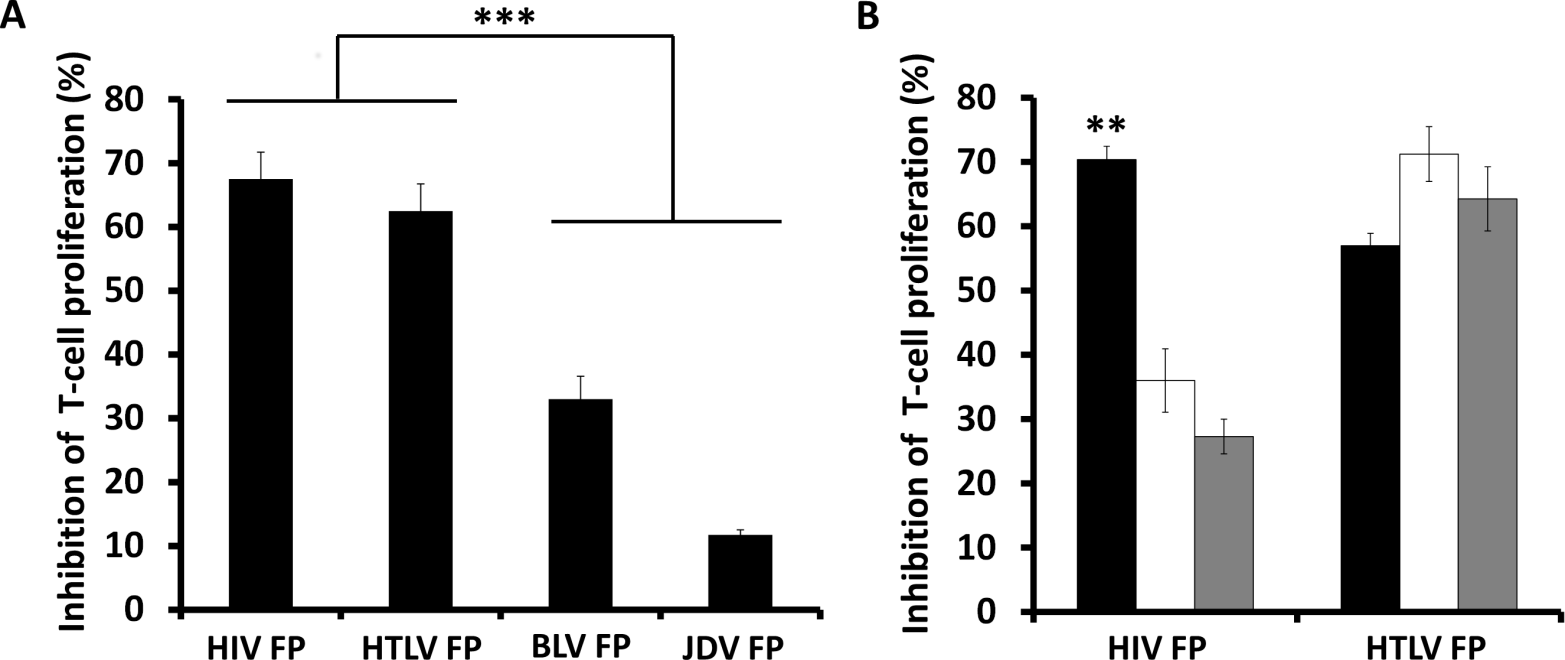
Inhibition of T-cell activation by the HTLV FP. (*A*) MOG_35-55_–antigen specific T-cells were activated by irradiated MOG_35-55_ presenting APCs, in the presence of several viral derived FPs at 10μM. The proliferative responses were assessed by H^3^-thymidine incorporation assay, and normalized to the proliferation of non-activated T-cells. The HTLV FP inhibits T-cell proliferation with the same potency as the HIV FP. The data is presented as mean inhibition of proliferation. n = 12. (*B*) MOG_35-55_–antigen specific T cells were activated by either (*i*) irradiated MOG_35-55_ presenting APCs (black), (*ii*) antibodies against CD3 and CD28 (white), or (*iii*) PMA and Ionomycin (gray) in the presence of the HTLV and HIV FPs at 10μM. Proliferation was assessed as described above. The HTLV FP inhibits T-cell proliferation induced either through the TCR or downstream from the TCR with equal potencies. The data is presented as mean inhibition of proliferation. n = 12. One-way ANOVA was used for statistical analysis. **P<0.01;***P<0.001.

To examine whether other viruses can utilize their FPs to interfere with T-cell activity we initially investigated the immunosuppressive ability of the HTLV FP on primary C57BL/6J mMOG(35–55)-specific T-cells upon activation by antigen presenting cells (APCs). We compared this activity to the well characterized HIV FP and to the bovine leukemia virus (BLV) and Jembrana disease virus (JDV) FPs. BLV and JDV are HTLV and HIV equivalents in cattle, respectively (Table 1). The HTLV FP was found to inhibit T-cell proliferation with equal potency to that of HIV’s FP and significantly stronger than the BLV FP. The JDV FP showed no inhibitory activity (Fig 1A). The HTLV FP was not toxic up to 4-fold higher than the concentrations used in this study (S1A Fig), even when viability was measured following 72 hours incubation of cells with the peptide (S1B Fig).

**Table 1:** Designation, sequence, and origin of peptides used in this study.

Designation	Sequence ^a^^,^ ^b^	Origin
**HTLV FP**	AVPVAVWLVSALAMGAGVAGGITGSMSLASGKS	HTLV-1
**HTLV Scr**	ASKAASASPVSAGVTGIGVLMLGVVAMGWASLG	HTLV-1
**HTLV FP**_**5-13**_	AVWLVSALA	HTLV-1
**HTLV FP**_**9-22**_	VSALAMGAGVAGGI	HTLV-1
**HTLV FP**_**14-22**_	MGAGVAGGI	HTLV-1
**HIV FP**	AVGIGALFLGFLGAAGSTMGARSMTLTVQARQL	HIV-1
**HIV FP**_**5-13**_	GALFLGFLG	HIV-1
**BLV FP**	VAALTLGLALSVGLTGINVAVSALSHQRLTSLI	BLV
**JDV FP**	AVGMVIFLLVLAIMAMTASVTAAATLVKQHATA	JDV

^a^ All peptides were synthesized using the F-moc solid phase method and purified by reverse phase HPLC.

^b^ The FP sequences are derived from the MT-2 isolate of HTLV-1, HXB2 isolate of HIV-1, Japanese isolate of BLV-1 and Tabanan/87 isolate of JDV.

T-cells can be activated *in-vitro* either directly through TCRα and β by antigen presentation, downstream to TCRα and β using antibodies against CD3 and CD28 or downstream to the entire TCR complex using the PKC activator PMA together with the Ca^+2^ ionophore Ionomycin [8, 9]. To examine where in the TCR signaling cascade the HTLV FP exerts its inhibitory activity, we activated T-cells using either APCs, CD3 and CD28 antibodies or PMA and Ionomycin. The HTLV FP inhibited T-cell proliferation induced at all three levels of activation with equal potency, while inhibition by the HIV FP that specifically targets the TCRα subunit, decreased significantly when activation was downstream of TCRα and β (Fig 1B), as previously reported [11]. This indicates that in contrast to HIV-1’s FP, the HTLV FP does not inhibit T-cell activation by targeting the TCR complex.

Following the previous results we tested HTLV FP specificity by assessing its inhibitory activity on activated macrophages. Primary mouse bone marrow derived macrophages (BMDM) were isolated, grown and stimulated using LTA, LPS, or PAM3CSK4, toll like receptor (TLR) 2/6, 4/4, and 2/1 ligands, respectively. The effect of HTLV FP treatment on TNFα and IL6 secretion was measured by ELISA. The HTLV FP had no effect on cytokine secretion from primary mouse BMDM (S2 Fig), demonstrating that the peptide selectively inhibits T-cell activation.

### Th1-specific cytokines transcription and secretion is reduced by the HTLV FP whereas Th2-specific cytokines are elevated

**Fig 2:**
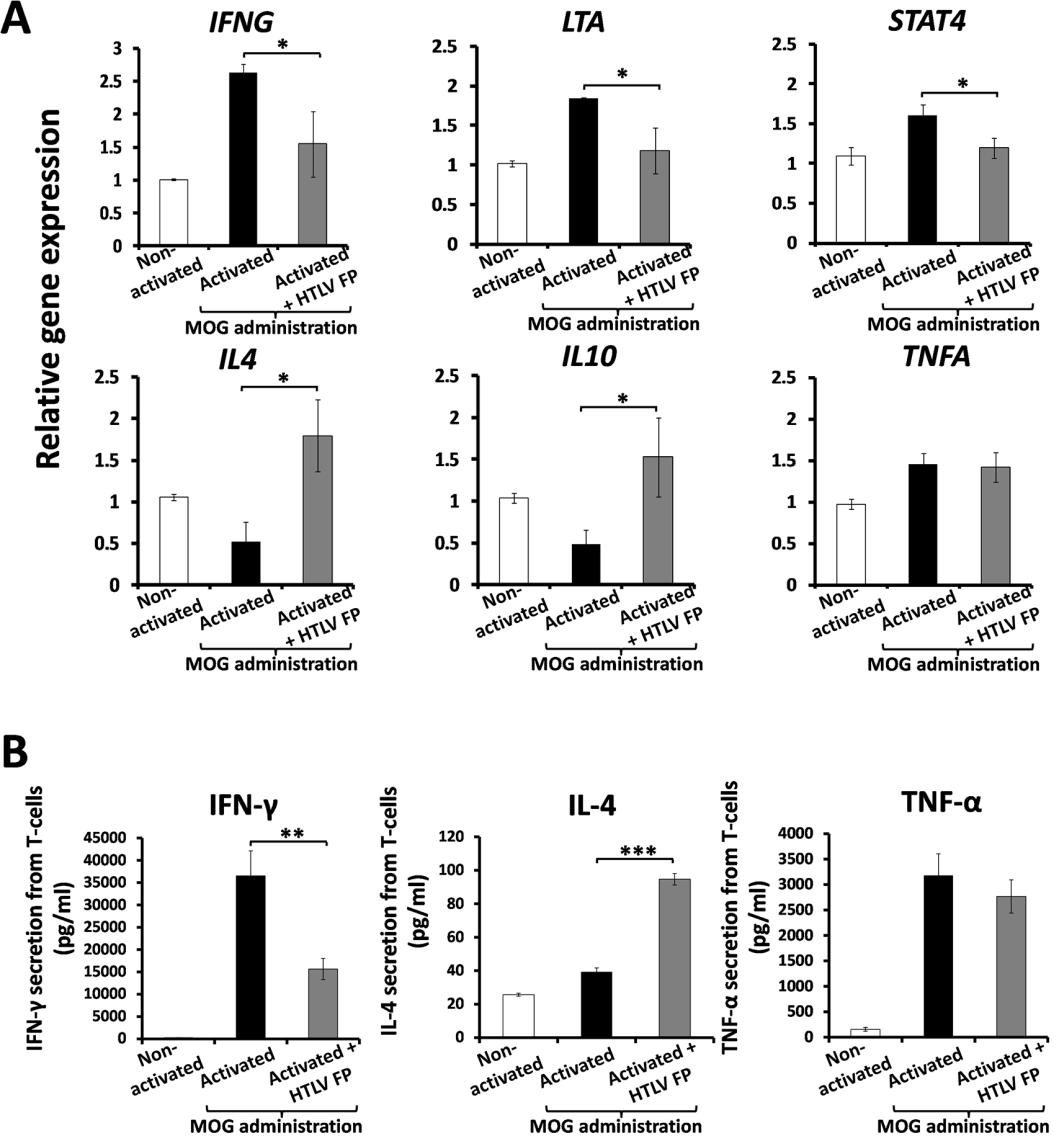
Modulation of Th1/Th2 gene expression and cytokine secretion from activated T-cells by the HTLV FP. MOG_35-55_–antigen specific T-cells were activated by irradiated MOG_35-55_ presenting APCs, in the presence of the HTLV FP at 10μM. (*A*) Total RNA was isolated 24 hours following activation and mRNA expression levels were determined by qRT-PCR. The values of each gene were normalized to Rpl13a as a housekeeping control. The HTLV FP reduces mRNA expression of Th1 specific genes (*IFNG*, *LTA*, and *STAT4*) and elevates mRNA expression of Th2 specific genes (*IL4* and *IL10*). The data is presented as arbitrary units. n≥3. (*B*) Media was collected 24 hours following activation and secretion of cytokines was measured by ELISA assay. IFN-γ secretion is inhibited, IL4 secretion is elevated and TNFα is not affected by the HTLV FP. n = 12. One-way ANOVA was used for statistical analysis. *P<0.05; **P<0.01;***P<0.001.

To further characterize the inhibitory mechanism of the HTLV FP, we examined its effect on the expression level of several Th1 and Th2 specific genes that are transcribed upon T-cell activation [33, 34]. C57BL/6J mMOG(35–55)-specific primary T-cells were activated using APCs. RNA was extracted 24hr following activation and mRNA levels were determined using RT-qPCR. The HTLV FP reduced the mRNA levels of the Th1-specific genes *IFNG*, *LTA* and the Th1 key mediator *STAT4* [35, 36] (Fig 2A). On the other hand, the HTLV FP elevated the mRNA levels of the Th2-specific genes *IL4* and *IL10* (Fig 2A). Yet, Tumor necrosis factor α (TNF-α), that is expressed by both subsets [37], was not affected (Fig 2A).

To determine whether the observed changes in gene expression can be observed at the protein level, we performed ELISA for selected cytokines. C57BL/6J mMOG(35–55)-specific primary T-cells were activated using APC and supernatants were collected 24hr following activation. The HTLV FP inhibited IFN-γ secretion, elevated IL4 secretion and had no effect on TNF-α secretion from activated T-cells (Fig 2B), further corroborating our RT-qPCR results. These findings suggest that HTLV-1 might utilize its FP to restrict the T-cell antiviral immune response by downregulation of the Th1 and upregulation of the Th2 responses.

### MOG35-55-induced experimental autoimmune encephalomyelitis (EAE) is alleviated by administration of the HTLV FP

As the HTLV FP was shown to inhibit mMOG(35–55)-specific primary T-cell activation *in-vitro* (Fig 1A and 1B) by downregulating their Th1 response (Fig 2), we examined inhibition of pathogenic MOG(35–55)-specific T-cells *in-vivo* by the HTLV FP. We tested this in an EAE model, which is a widely used mouse model that mimics chronic MS in humans [38, 39] and is considered a CD4^+^ Th1-mediated autoimmune disease [40, 41]. We performed an initial experiment in which C57BL/6J mice were immunized with MOG35-55/CFA for EAE induction and were either treated with a single dose of HTLV FP (1mg/kg) or vehicle. Clinical manifestation of EAE for vehicle-treated mice was first observed at 8 days post immunization (DPI), reaching a severe disease at 10 DPI, and was accompanied with a substantial loss of weight (S3A and S3B Fig). Moreover, clinical severity was correlated to reduced locomotion as a result of hind limb ataxia or paralysis (S1 and S2 Movies). In contrast, HTLV FP treated mice showed only mild clinical symptoms that were first observed at 11 DPI and were followed only by minor weight fluctuations (S3A and S3B Fig). Additionally, most of the HTLV FP treated mice exhibited normal locomotory behavior throughout the experiment (S3 and S4 Movies). As disease manifestation in vehicle treated mice was relatively severe, experiment was terminated at 14 DPI due to institutional animal care and use committee (IACUC) limitations and requirements.

**Fig 3:**
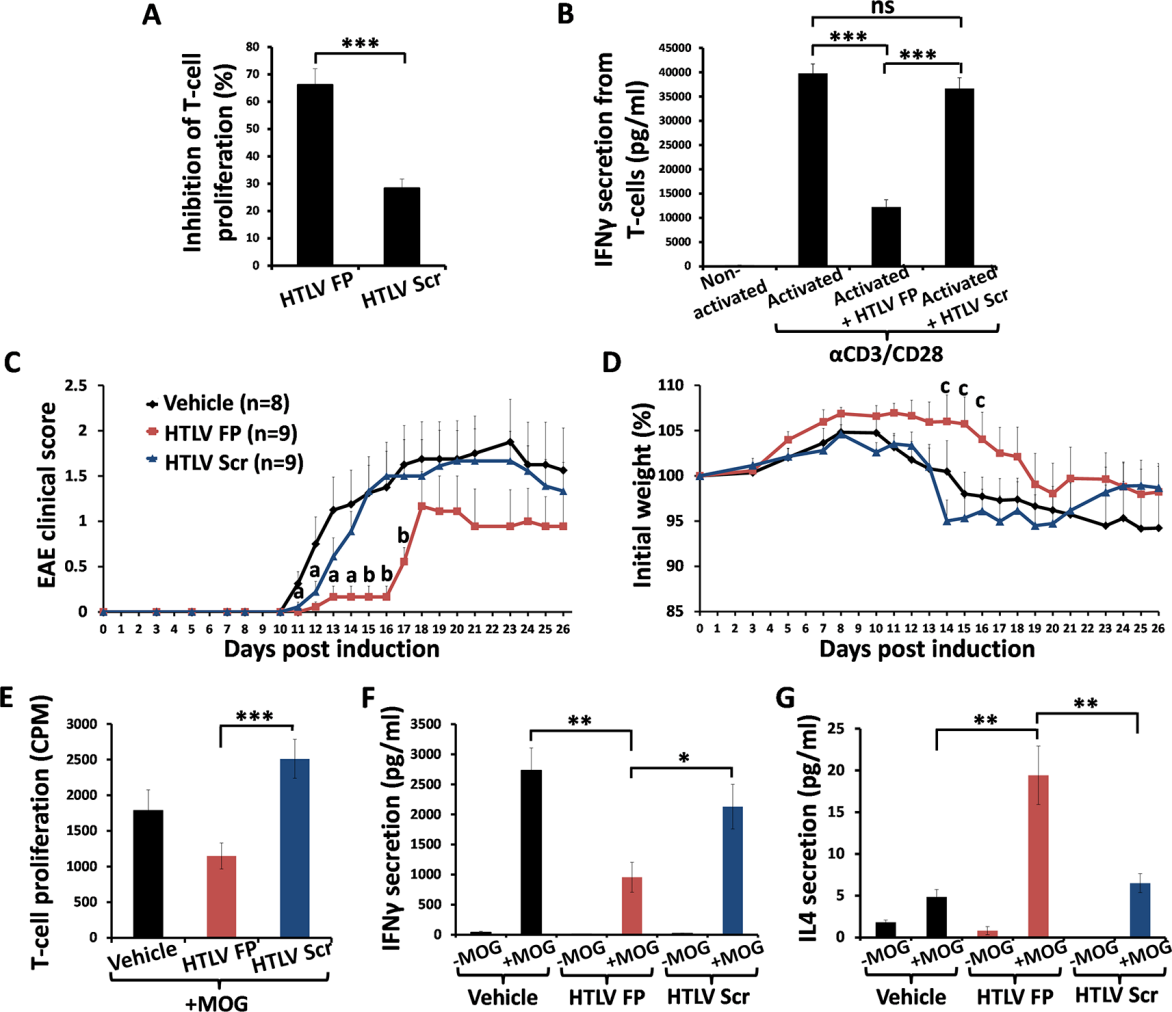
Administration of HTLV FP alleviates MOG35-55-induced EAE. (*A-B*) MOG_35-55_–antigen specific T-cells were activated by irradiated MOG_35-55_ presenting APCs, in the presence of HTLV FP or HTLV Scr at 10μM. (*A*) The proliferative responses were assessed by H^3^-thymidine incorporation assay, and normalized to the proliferation of non-activated T-cells. The data is presented as mean inhibition of proliferation. n = 12. (*B*) IFN-γ was measured by ELISA assay. n = 12. (*C-D*) EAE was induced in C57BL/6 female mice that were treated with a single dose of HTLV FP, HTLV Scr or vehicle. Two indexes to measure clinical disease severity are displayed. (*C*) Direct clinical measurement of EAE phenotype in a 5-point scale with increased disease symptoms correlating with higher score value. The data is presented as mean EAE clinical score. (*D*) Mice were weighed the day before EAE induction, and the change (as a percentage) in weight was recorded. The data is presented as mean change from the initial weight. One-way ANOVA was used for statistical analysis. ^**a**^ HTLV FP ≠ Vehicle; ^**b**^ HTLV FP ≠ Vehicle & HTLV Scr; ^**c**^ HTLV FP ≠ HTLV Scr. ^**a,b,c**^*P*<0.05. (*E*) Spleenocytes were harvested at 26 DPI and stimulated using MOG35-55. The proliferative responses were assessed by H^3^-thymidine incorporation assay and normalized to the proliferation of non-activated T-cells. The data is presented as counts per minute (CPM). n = 7. (*F-G*) Spleenocytes were harvested at 26 DPI and stimulated using MOG35-55. Media was collected 48 hours following activation and secretion of IFN-γ and IL4 was measured by ELISA assay. HTLV FP treatment inhibits IFN-γ and elevates IL4 secretion from MOG35-55-reactive T-cells. n>8. One-way ANOVA was used for statistical analysis. *P<0.05;**P<0.01;***P<0.001; ns, not significant.

In order to examine whether the HTLV FP sequence is crucial for its inhibitory activity we synthesized a scrambled HTLV FP peptide (HTLV Scr), consisting of the same amino acid composition and length as the HTLV FP (Table 1). The effect of both peptides on mMOG(35–55)-specific primary T-cell activation was compared. The HTLV FP inhibited both T-cell proliferation and IFN-γ secretion with higher potency than the HTLV Scr (Fig 3A and 3B), demonstrating that the HTLV FP sequence is critical for its inhibitory activity.

We then performed an additional EAE experiment in which mice were treated with a single dose of HTLV FP (1mg/kg), HTLV Scr (1mg/kg) or vehicle. EAE clinical signs of vehicle and HTLV Scr treated groups ascent between 11 to 16 DPI, a period during which the HTLV FP treated mice showed only mild clinical symptoms and weight loss (Fig 3C and 3D). Moreover, most of the HTLV FP treated mice exhibited better locomotory behavior than the HTLV Scr treated mice throughout the experiment (S5 and S6 Movies). As disease manifestation was milder compared to the previous experiment, we were able to monitor animals for a longer period of time. In order to determine whether the reduction in EAE severity upon HTLV FP treatment actually results from downregulation of pathogenic MOG35-55-reactive T-cells, spleens were harvested at 26 DPI, cultured *ex-vivo*, and stimulated using MOG35-55. Stimulation with MOG35-55 resulted in a T-cell proliferative response that was significantly lower in HTLV FP treated spleenocytes compared to HTLV Scr treated spleenocytes (Fig 3E). In addition, HTLV FP treatment resulted in significantly lower IFN-γ secretion and significantly higher IL4 secretion compared to both vehicle and HTLV Scr treated groups (Fig 3F and 3G). These results demonstrate that the HTLV FP modulates antigen-specific T-cell activation *in-vivo* leading to a downregulation of Th1 and upregulation of Th2 responses.

### Primary human peripheral T-cell activation is inhibited by HTLV FP treatment

**Fig 4:**
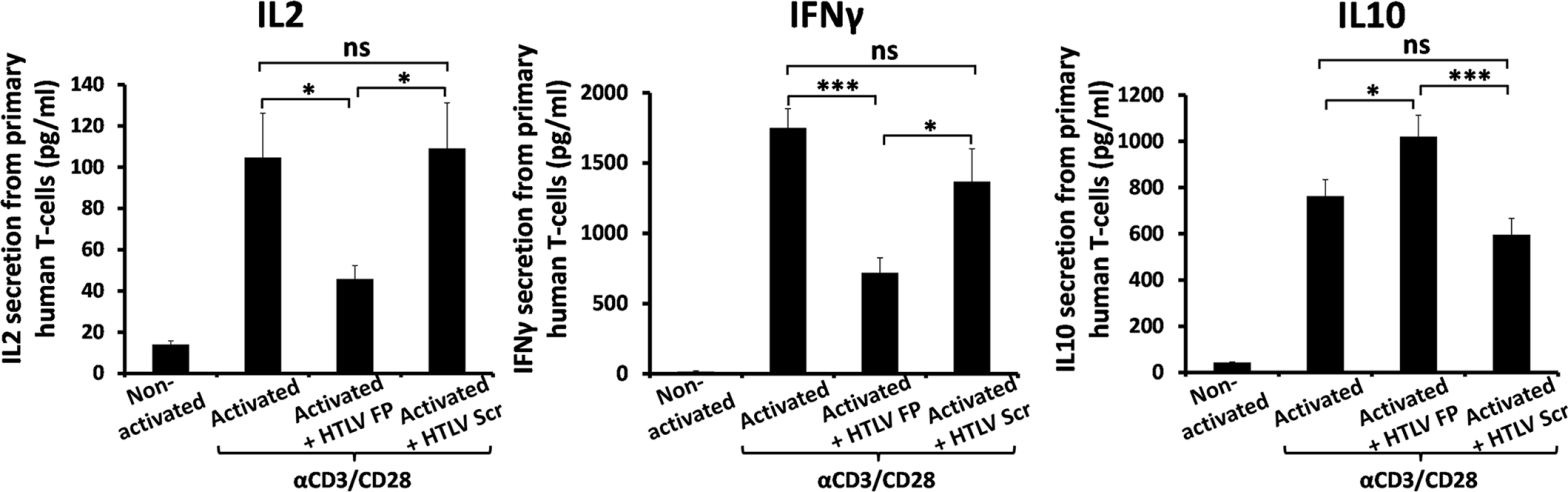
Primary human T-cell activation is inhibited upon HTLV FP treatment. Human peripheral T lymphocytes were isolated from whole blood and activated using CD3 and CD28 antibodies, in the presence of HTLV FP or HTLV Scr at 10μM. Media was collected 48 hours following activation and secretion of IL2, IFN-γ and IL10 was measured by ELISA assay. HTLV FP treatment inhibits IL2 and IFN-γ and elevates IL10 secretion from primary human peripheral T-cells. n = 12. One-way ANOVA was used for statistical analysis. *P<0.05; ***P<0.001; ns, not significant.

As HTLV-1 is a T-cell infecting human pathogen [17] and since in this study we show that the HTLV FP inhibits T-cell activation in mice both *in vitro* and *in vivo*, we aimed to determine whether this inhibition would apply to human T-cells as well. Hence, human peripheral T lymphocytes were isolated from Peripheral blood mononuclear cells (PBMCs), cultured *ex-vivo* and activated using CD3 and CD28 antibodies. The effect of HTLV FP treatment on secretion of the T-cell activation marker IL2 was measured by ELISA, as well as the Th1- and Th2-specific cytokines IFN-γ and IL10, respectively. HTLV FP treatment resulted in a significant reduction in IFN-γ and IL2 secretion and an elevation in IL10 secretion, while treatment with HTLV Scr had no effect on T-cell activation (Fig 4). These results demonstrate that the modulatory activity of the HTLV FP is not limited to mouse cells or to cells that recognize a certain antigen and further emphasize its sequence specificity.

### T-bet expression is reduced while Gata3 expression is elevated following HTLV FP treatment

**Fig 5:**
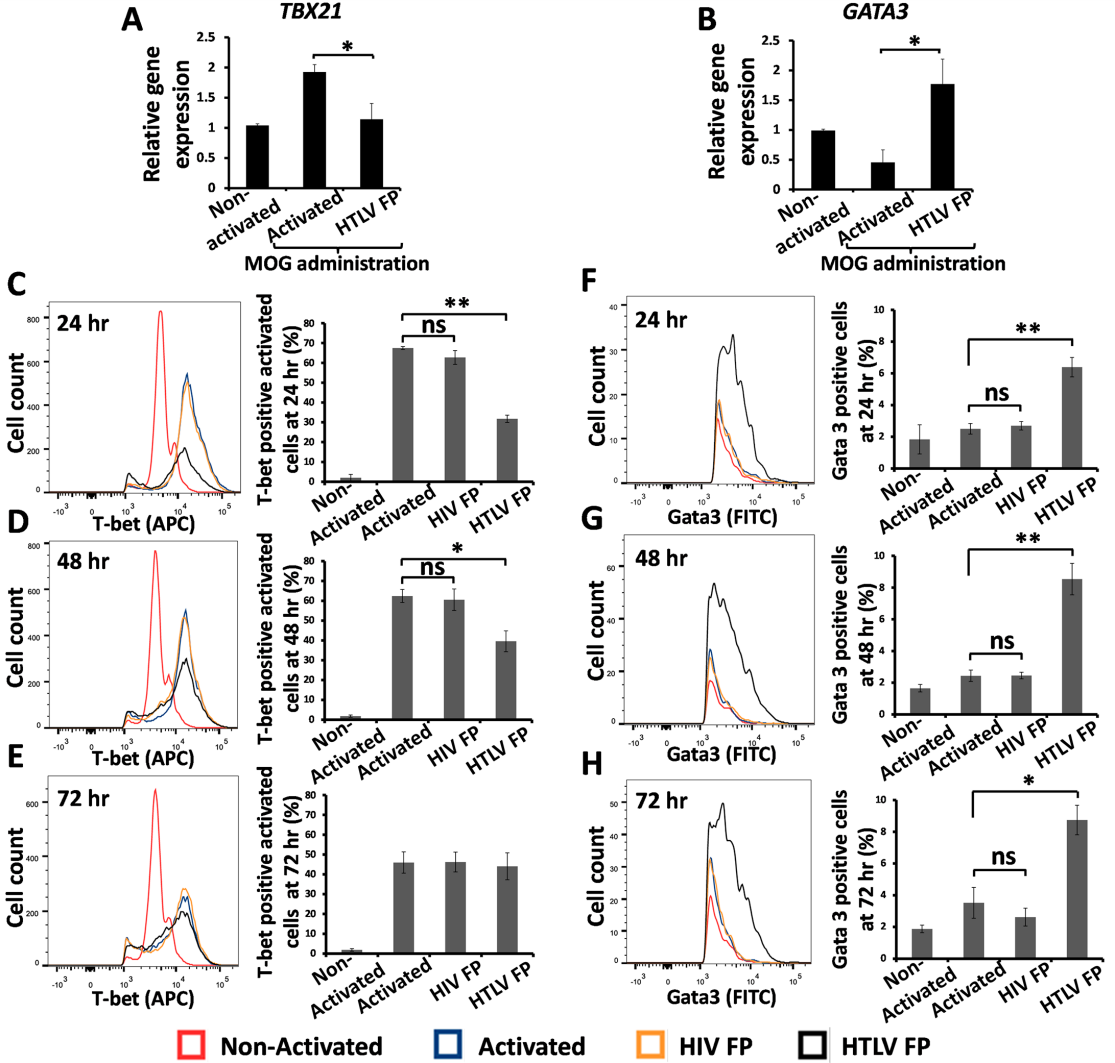
Reduced T-bet expression and elevated Gata3 expression induced by the HTLV FP. (*A-B*) MOG_35-55_–antigen specific T-cells were activated by irradiated MOG_35-55_ presenting APCs, in the presence of the HTLV FP at 10μM. Total RNA was isolated 24 hours following activation and mRNA expression levels were determined by qRT-PCR. The values of each gene were normalized to Rpl13a as a housekeeping control. The HTLV FP reduces *TBX21* (T-bet) mRNA expression and elevates *GATA3* mRNA expression. The data is presented as arbitrary units. n≥3. (*C-H*) MOG_35-55_–antigen specific T-cells were activated by irradiated MOG_35-55_ presenting APCs, in the presence of either HTLV or HIV FP at 10μM. Samples were fixed in 4% PFA and stained with anti T-bet-APC and anti Gata3-FITC antibodies (*C-D*) 24, (*E-F*) 48 and (*G-H*) 72 hours following activation. Each time point is represented as cell count vs. APC or FITC fluorescence histogram. Analysis was performed using LSR-II flow cytometer (BD) and FlowJo cell analysis software (FlowJo, LLC). The HTLV FP reduces T-bet expression and elevates Gata3 expression. n = 3. One-way ANOVA was used for statistical analysis. ns, not significant; *P<0.05;** P<0.*01*.

To determine whether the transition in cytokine pattern driven by the HTLV FP is indicative of a Th1 to Th2 transition we examined the expression of Th1 and Th2 specific transcription factors, T-bet and Gata3 respectively. C57BL/6J mMOG(35–55)-specific primary T-cells were activated using APCs following HTLV FP treatment. RNA was extracted 24hr following activation and mRNA levels were determined using RT-qPCR. HTLV FP treatment resulted in a reduced *TBX21* (T-bet) expression and elevated *Gata3* expression (Fig 5A and 5B). We next examined the expression of these genes at the protein level via FACS analysis. C57BL/6J mMOG(35–55)-specific primary T-cells were activated using APCs following either HTLV FP or HIV FP treatment, collected 0, 24, 48 and 72 hours following activation and stained for T-bet and Gata3. Initially, we gated on T-bet expressing lymphocytes (S5A Fig). Since our mMOG(35–55)-specific primary T-cells express basal level of T-bet that is elevated upon activation, we focused on the activated subset of lymphocytes (S5B Fig). The HTLV FP reduced T-bet expression 24 and 48 hours following activation, while the HIV FP had no effect on T-bet expression (Fig 5C and 5D). However, after 72 hours no difference was observed (Fig 5E), though T-bet expression of activated cells diminished in comparison to 24h and 48h (S6 Fig). When gating on Gata3 expressing cells (S7 Fig), we found that the HTLV FP elevated Gata3 expression 24, 48 and 72 hours following activation while the HIV FP had no effect on Gata3 expression (Fig 5F–5H). Taken together, these results suggest that HTLV FP administration downregulates the Th1 response leading to a more Th2-like response.

### The FP_5-13_ is the immune modulatory segment within the HTLV FP

**Fig 6:**
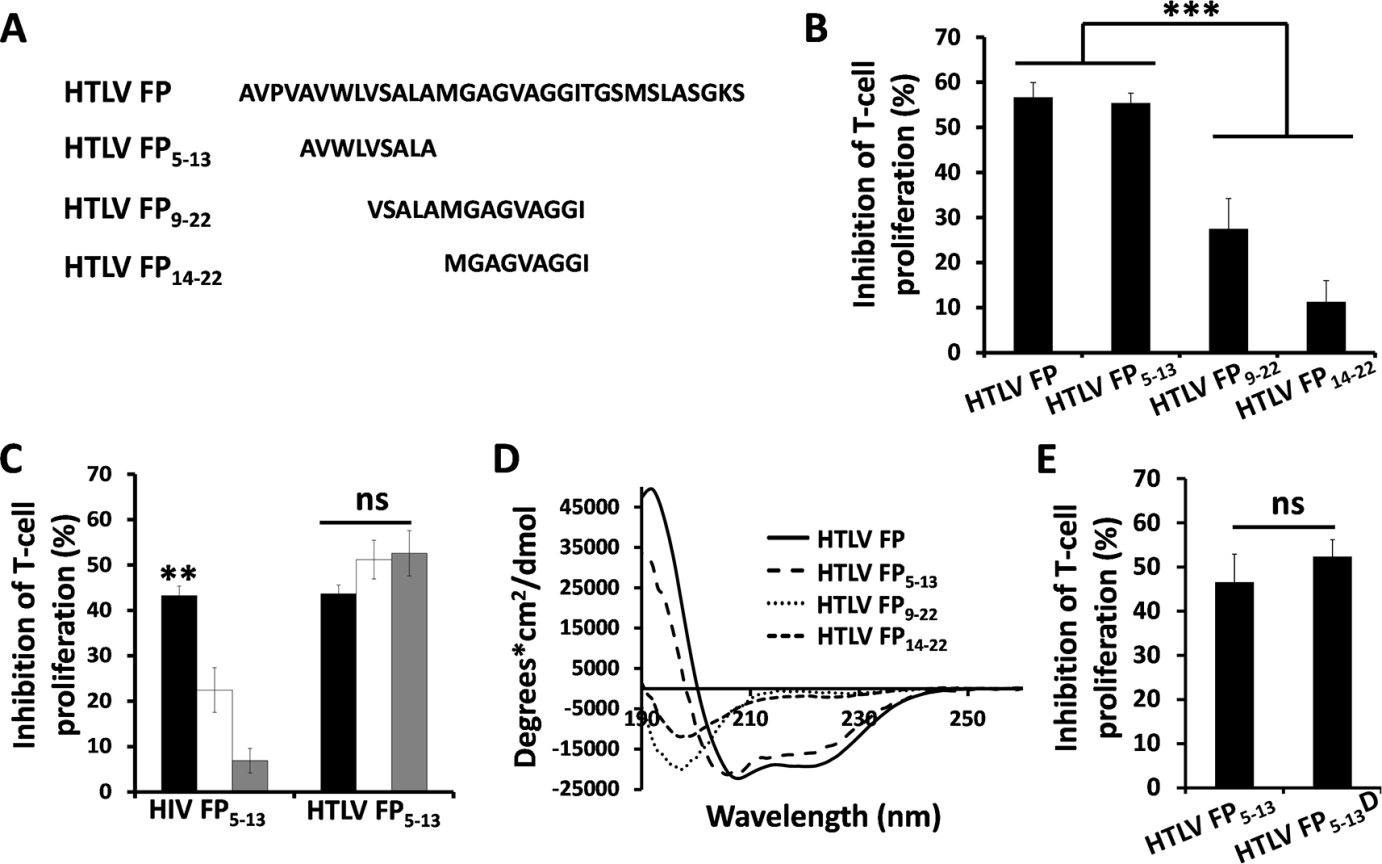
Detection of the immune modulatory region within the HTLV FP. (*A*) Sequences of shorter peptides derived from the full length HTLV FP. (*B*) MOG_35-55_–antigen specific T-cells were activated by APCs in the presence of HTLV FP derived peptides at 10μM and proliferative responses were assessed as described above. The HTLV FP and HTLV FP_5-13_ inhibit T-cell proliferation with higher potency than HTLV FP_9-22_ and HTLV FP_14-22_. The data is presented as mean inhibition of proliferation. n = 12. (*C*) MOG_35-55_–antigen specific T cells were activated by either (*i*) irradiated MOG_35-55_ presenting APCs (black), (*ii*) antibodies against CD3 and CD28 (white), or (*iii*) PMA and Ionomycin (gray) in the presence of HTLV FP_5-13_ and HIV FP_5-13_ at 10μM. Proliferative responses were assessed as described above. The HTLV FP inhibits T-cell proliferation induced either through the TCR or downstream from the TCR with equal potencies. The data is presented as mean inhibition of proliferation. n = 12. (*D*) Secondary structures of HTLV FP derived peptides as revealed by CD spectroscopy. CD spectra were measured at 25μM in 5mM HEPES buffer containing 1% lyso-phosphatidylcholine (LPC). The HTLV FP and HTLV FP_5-13_ exhibit a typical α-helical curve while the HTLV FP_9-22_ and HTLV FP_14-22_ exhibit a typical random coil. (*E*) MOG_35-55_–antigen specific T-cells were activated by APCs in the presence of HTLV FP_5-13_ D/L enantiomers at 10μM and proliferative responses were assessed as described above. Both HTLV FP_5-13_ enantiomers inhibit T-cells proliferation with equal potencies. The data is presented as mean inhibition of proliferation. n = 12. One-way ANOVA was used for statistical analysis. *P<0.05;**P<0.01;***P<0.001.

In order to detect the active segment within the HTLV FP, three peptides, designated HTLV FP_5-13_, HTLV FP_9-22_ and HTLV FP_14-22_ (Table 1), were synthesized based on the network protein sequence (NPS) secondary consensus prediction method [42] (Fig 6A). The HTLV FP_5-13_ peptide encompasses the helical predicted section of the HTLV-1 FP (S8 Fig) and is located at the same region previously found to be active segment of the HIV FP, both at the 5–13 amino acid section [12]. The HTLV FP_9-22_ peptide consists of two consecutive repeats of the known GxxxG-like dimerization motif [43, 44], while the HTLV FP_14-22_ peptide contains only one. These HTLV FP derived peptides were then examined for their ability to inhibit T-cell proliferation. This analysis revealed that both HTLV FP_1-33_ and FP_5-13_ are significantly more active compared to the FP_9-22_ and FP_14-22_ (Fig 6B). In order to compare the ability of the HIV FP_5-13_ and HTLV FP_5-13_ to suppress the induction of T-cell activation at different steps of the TCR signaling cascade, C57BL/6J mMOG(35–55)-specific primary T-cells were activated using either APC, CD3 and CD28 antibodies or PMA and Ionomycin. Similar to the HTLV FP, the HTLV FP_5-13_ inhibited all three levels of activation (Fig 6C). Peptides were not toxic to T-cells at concentrations used in this study (S1A Fig). In contrast, the activity of the HIV FP_5-13_ significantly diminished when T-cells were activated using CD3 and CD28 antibodies or PMA and Ionomycin (Fig 6C). The secondary structures of the peptides were then determined in a membrane mimetic environment using circular dichroism (CD) (Fig 6D) and analyzed for structure proportions using CDNN. Both the HTLV FP and HTLV FP_5-13_ exhibited an α-helical structure while the HTLV FP_9-22_ and HTLV FP_14-22_ were found to be random coils (Table 2). This result suggests that the loss of inhibitory activity by the HTLV FP_9-22_ and FP_14-22_ might be due to loss of secondary structure.

**Table 2:** CD spectra analysis of the HTLV FP derived peptides by the CDNN secondary structure analysis program.

	α-Helix	Antiparallel β-Sheet	Parallel β-Sheet	β-turn	Random-coil
**HTLV FP**	68.6	2.1	3.3	13	13
**HTLV FP**_**5-13**_	54	6.7	5.2	16.8	17.3
**HTLV FP**_**9-22**_	8.5	30.4	11.7	16.8	32.6
**HTLV FP**_**14-22**_	11.2	27.7	11.7	17.1	32.5

Peptides were dissolved in HEPES buffer with 1% LPC and secondary structure was obtained by circular dichroism spectroscopy (190–260 nm) and analyzed via CDNN (Applied Photophysics Ltd). Values represent the relative amount of structure out of 100%.

Next we aimed to determine whether T-cell inhibition by the HTLV FP_5-13_ occurs within the membrane. For that purpose we utilized a D-enantiomer form of the HTLV FP_5-13_ (designated HTLV FP_5-13_D) as interactions of peptides and proteins in the membrane have been shown to be chirality independent [45–47]. We activated our C57BL/6J mMOG(35–55)-specific primary T-cells with APCs and examined their proliferative response following treatment with HTLV FP_5-13_ and HTLV FP_5-13_D. Both peptides inhibited T-cell proliferation with the same potency (Fig 6E), suggesting that their active site is within the membrane. Overall, these results indicate that the HTLV FP_5-13_ is the immune modulatory segment of the HTLV FP and that it acts as an α-helix in the membrane.

## Discussion

HIV-1 utilizes the FP of its gp41 fusion protein to downregulate T-cell activation [11]. Yet, it is unknown whether this ability is shared by other viruses. We utilized the FP of the CD4^+^ T-cell infecting retrovirus HTLV-1 to explore whether other viral FPs might exhibit immune modulating properties. We reveal that the HTLV-1 gp21 FP is a potent suppressor of T-cell activation, demonstrated by its ability to reduce the onset of the EAE multiple sclerosis (MS) model in mice. Comparing both HIV-1 and HTLV FPs reveals that in contrast to HIV-1, the HTLV FP’s inhibitory effect occurs downstream of the TCR complex and is associated with a decrease in Th1 responses and an elevation in Th2 responses.

Activation of T-cells can be induced *in-vitro* by antigen presentation either through the TCR itself, downstream of the TCR using CD3 and CD28 antibodies or downstream from the entire TCR complex via PMA and Ionomycin [8, 9]. Here we found that in contrast to the HIV FP, the HTLV FP does not exert its inhibitory effect by targeting the TCR complex. This raises a question regarding the specificity of HTLV-1’s FP inhibitory activity to T-cells, yet, the peptide had no effect on the activation level of mouse primary bone marrow derived macrophages, supporting its specificity T-cells.

In order to elucidate HTLV FP’s mechanism of action, we examined its effect on mRNA expression and cytokine secretion levels of several Th1 and Th2 specific genes that are transcribed upon T-cell activation [33, 34]. The HTLV FP inhibited expression and secretion of Th1-specific cytokines that are crucial for the T-cell antiviral response [48, 49], yet, elevated Th2-specific cytokines [50, 51]. These findings suggest a shift in the Th1/Th2 balance promoted by the HTLV FP. A Th1 response evokes cell-mediated immunity, therefore crucial for the eradication of intracellular pathogens such as viruses. On the other hand a Th2 response controls humoral immunity and evokes antibody responses, which govern the elimination of extracellular pathogens [52]. Skewing the Th1/Th2 balance towards a Th2 response is beneficial for viral persistence within its host and several viruses have been shown to utilize this immune modulation strategy [53, 54]. This is also evident by the findings that some antiviral compounds exert their activity by increasing the Th1 response [55]. Studies indicate that HTLV-1 infection induces IFN-γ production that is aimed to eradicate the virus [56, 57]. As the HTLV FP is exposed during membrane fusion [32], our data suggests that the virus might have the ability to utilize this gp21 region to antagonize this initial anti-viral immune response, thus to better persist within its host. This is in line with evidence showing that HTLV-1 harbors Th1 suppressing factors [58]. Additionally, the ISD that was previously identified within retroviral envelope proteins [25], including the HTLV-1 gp21, has been reported to decrease Th1 and increase Th2 cytokine production [59]. As in the case of the ISD, HTLV FP treatment inhibited IL2 secretion [60]. The ISD is proposed to induce this immune modulation by elevation of cAMP concentration and by inhibition of protein kinase C (PKC) [59, 61]. In this study we show that the HTLV FP significantly inhibits T-cell activation through PMA (PKC activator) and Ionomycin [11], indicating some similarities between the HTLV FP and the ISD derived peptide mechanisms of action. Yet, additional work is required in order to elucidate the exact mechanism of HTLV FP immune suppression.

Th1- and Th2-responses are orchestrated by the specific transcription factors T-bet and Gata3, respectively [62–64]. Therefore, changes in the expression level of these proteins seen in this study further suggest a shift in the Th1/Th2 balance. Yet, though significantly elevated, the fold change of Gata3 expression levels in activated versus non-activated cells was low compared to T-bet as seen by FACS analysis. This suggests that the HTLV FP is not directly elevating Gata3 expression but rather inhibiting T-bet. Since Gata3 expression is negatively regulated by T-bet expression and vice versa [63, 64], it is plausible that the remarkably sharp decrease in T-bet expression is sufficient to cause a prolonged elevation in Gata3 expression. This experimental evidence suggests that by downregulating T-bet expression the HTLV FP disrupts the Th1/Th2 balance, thus elevating Gata3 expression. Yet, other T-cell subsets such as T regulatory cells (Treg) are infected by HTLV-1 [56, 65]. Interestingly, although the development and function of Treg is governed by the master regulator FoxP3 [66], this T-cell subset has been shown to express Gata3 as well [67]. Yet, in contrast to its inhibitory effect on T-bet, Gata3 has been shown to be crucial for Treg function and homeostasis by enhancing FoxP3 expression [68–70] as reviewed in [71]. Interestingly, an HTLV-1 derived factor has been shown to induce CCR4 expression through induction of Gata3 in Treg, promoting T-cell migration and proliferation [72]. Since cell-free HTLV-1 virions are poorly infectious [73, 74] the virus mainly spreads from cell to cell through virological synapses [75]. Thus, promoting T-cell migration is of crucial importance for viral transmission and propagation as it allows infected cells to infiltrate healthy tissues eventually supporting transmission from infected to non-infected cells. Overall, HTLV-1 might utilize its FP to modulate the activity of different T-cell subsets through elevation of Gata3 expression in order to support its persistence within hosts.

As the HTLV FP was shown to downregulate Th1-responses *in-vitro*, we examine its effect on the induction of a Th1-mediated autoimmune disease *in-vivo*. EAE is induced by immunization with myelin peptides, such as MOG(35–55), emulsified in CFA, yet, it can be induced by adoptive transfer of myelin-specific CD4^+^ Th1 cells into naïve recipient mice as well [76–82]. In addition, Stat4 and T-bet, transcription factors in the Th1 differentiation pathway, have been shown to be essential for EAE induction [81, 83–85]. In this study, we demonstrate that the HTLV FP downregulates the transcription of Stat4 and T-bet mRNA, as well as inhibiting mMOG(35–55)-specific primary T-cell activation *in-vitro*, making EAE an ideal *in-vivo* model. Here we demonstrate that inhibition of EAE clinical signs by the HTLV FP specifically results from downregulation of pathogenic MOG35-55-reactive T-cells. Interestingly, in humans a small percentage of HTLV-1 infected individuals develop a chronic neuroinflammatory disease termed HTLV-1-associated myelopathy/tropical spastic paraparesis (HAM/TSP) [86] that has some pathological similarities to MS. In both cases, lymphocytes that infiltrate the CNS secrete pro-inflammatory cytokines such as IFN-γ and TNF-α [87–89] that can induce neurotoxicity at high concentrations [90, 91]. This results in spinal lesions that initially lead to muscular weakness in the lower limbs [87]. Additionally, soluble TNF-α receptor has been suggested as a common marker for monitoring the progression of these diseases [88] that are both characterized by Th1 predominance [41, 87]. In light of our results, the fact that only a small percentage of HTLV-1 infected individuals eventually develop HAM/TSP might be partially attributed to the neuro-protective nature of the HTLV FP, demonstrated here by its ability to specifically downregulate IFN-γ secretion from pathogenic T-cells in EAE and to alleviate and delay disease onset.

Next, we aimed to identify the immune modulatory region within the HTLV FP. NPS secondary structure prediction analysis [42] predicted the HTLV FP_5-13_ to be alpha helical. This region was found to be alpha helical in CD analysis and inhibited T-cell activation in contrast to the non-alpha helical regions of the HTLV FP. As we assume that the HTLV FP functions in the membrane, it is likely that an alpha helical structure would support its activity as interactions within the membrane are typically mediated by helix-helix interactions [92–94] through dimerization motifs [95–97], such as GxxxG [30, 98–102]. Hence, we concluded that the 5–13 region is the active segment within the HTLV FP. Interestingly, this is the same region previously identified as HIV-1’s FP active segment [12]. Since HIV-1 and HTLV-1 FPs completely differ in sequence, it seems that possibly through convergent evolution both viruses have obtained the ability to downregulate T-cell activation using the same region of their fusion protein.

The membrane environment holds unique characteristics that allow protein-protein interactions that would not be energetically favored in soluble environment [45], such as interactions between L- and D-enantiomer proteins [47]. Such chirality-independence has been utilized for inhibition of HIV cell-cell fusion by the HIV FP D-enantiomer [46], inhibition of T-cell activation by gp41’s loop derived peptides [103] and for inhibition of Tar receptor mediated chemotaxis in *E*.*coli* [47]. In these cases, the L- and D-enantiomers had the same potency. Since FPs are membranotropic regions of viral fusion proteins [104], and as HIV’s FP was specifically shown to target the transmembrane domain of the TCR within the membrane [11], we utilized a D-enantiomer form of the HTLV FP_5-13._ As both L- and D- peptides were found to inhibit T-cell proliferation with the same potency, we concluded that their active site is situated within the membrane. This is in line with evidence showing that upon binding of the envelope’s surface subunit gp46 to its cellular receptors, the HTLV-1 gp21 FP is exposed and then binds and perturbs the membrane eventually leading to fusion [32, 105].

In summary, our findings indicate that FP mediated T-cell immunosuppression is not unique to HIV, and suggest that it might be a more widespread immune evasion strategy utilized by viruses. Yet, it seems that the HTLV-1 and HIV-1 FPs exert their inhibitory activity on T-cells through different mechanisms thus demonstrating that there are distinct manners by which T-cell activation can be overcome. Our findings demonstrate that the HTLV FP has the capacity to downregulate Th1-mediated antiviral immune response, suggesting that the virus might utilize it for T-cell modulation during fusion. Yet, additional studies using HTLV-1 particles or HTLV-infected cells are required in order to be more conclusive. As the HTLV-1 gp21 FP is known to mediate membrane fusion [106], its ability to modulate T-cell activity highlights how viruses have evolved to alter different cellular processes with limited repertoire of proteins.

## Methods

### Mice

C57Bl/6J mice were purchased from Jackson Laboratories (Bar Harbor, ME, USA). All mice were 2–3 month-old when used in the experiments.

### Mouse antigen-specific T-cell isolation and culture

Antigen-specific T-cells were selected in-vitro [107] from primed lymph node cells derived from C57Bl/6J mice that had been immunized 9 days before with antigen (100μg myelin peptide, MOG35-55) emulsified in complete Freund’s adjuvant (CFA) containing 150μg Mycobacterium tuberculosis (Mt) H37Ra (Difco Laboratories, Detroit, MI). T-cells were maintained in-vitro in medium containing 500 ml RPMI Ca/Mg + heat inactivated FCS (10% final) + 5 ml 200 mM L-Glu (2 mM final) +5 ml 100 M Na pyruvate (1 mM final) + 5 ml Pen Strep antibiotics + 5 ml Eagle-MEM (Biological Industries, Ref 01-340-1B) + interleukin-2 (IL-2), with alternate stimulation with the antigen every 14 days.

### Bone marrow derived macrophage isolation

Mouse Femora and tibiae BM cells were isolated from C57Bl/6J mice and cultured in RPMI medium containing FBS (10%), L-glutamine (1%), sodium pyruvate (1%), Pen-strep (1%), and 10 ng/ml recombinant CSF-1 (Peprotech). At day 3, half the medium was replaced, and on day 7, cells were used for *in vitro* assay, in which 2*10^5^ cells were plated per well in a 24-well plate.

### Primary human peripheral T-cell purification

Human peripheral T lymphocytes were isolated from whole blood of healthy adult donors by dextran sedimentation and Ficoll (Sigma) gradient separation followed by depletion of B cells using nylon wool column (Unisorb), to which B cells were adsorbed. Cells were incubated in a complete RPMI growth medium (500 ml RPMI Ca/Mg + heat inactivated FCS (10% final) + 5 ml 200 mM L-Glu (2 mM final) +5 ml 100 M Na pyruvate (1 mM final) + 5 ml Pen Strep antibiotics) for more than 2 h, and then non-adherent cells were harvested and transferred to a new plate, resulting in ~90% CD3+ T lymphocytes. Cells were then used for in vitro assay, in which 105 cells were plated per well in a 96-well plate and activated using CD3 and CD28 antibodies.

### Peptide synthesis and purification

Peptides were synthesized using the F-moc solid phase method on Rink amide resin (0.65mmol/gr), as previously described [108]. The peptides were purified by reverse phase HPLC (RP-HPLC) to >95% homogeneity on a C4 or C2 column using a linear gradient of 20–70% acetonitrile in 0.1% trifluoroacetic acid (TFA) for 45 minutes. The peptides were subjected to ESI–MS (electrospray ionization mass spectrometry) analysis to confirm their composition.

### *In-vitro* T-cell proliferative response

Antigen-specific T-cells were plated onto round 96-well plates in medium containing RPMI-1640 supplemented with 2.5% fetal calf serum (FCS), 100 U/ml penicillin, 100 μg/ml streptomycin, 50μM β-mercaptoethanol, and 2mM L-glutamine. Each of the 96 wells contained 10^4^ T-cells, 5x10^5^ irradiated (25 gray) antigen presenting cells (APC), and 5μg/ml of MOG p35-55. In addition, the relevant peptide was added. In order to exclude interaction between the examined peptides and the MOG p35-55 antigen, we added the MOG p35-55 antigen to the APC in a test tube, and in a second test tube we added the examined peptides to the T-cells. After 1 hour, we mixed the APC with the T-cells and incubated them for 48h in a 96 well round bottom plate. Then T-cells were pulsed with 1μCi (H^3^) thymidine, with a specific activity of 5.0 Ci/mmol, for 24 hours, and (H^3^) thymidine incorporation was measured using a 96-well plate beta-counter. The mean cpm ± SD was calculated for each quadruplicate. In several experiments, cells were activated with pre-coated CD3 and CD28 antibodies (LEAFTM purified anti mouse clones 145-2-C11 and 37.51, respectively from Biolegend) at final concentration of 2μg/ml, or 50ng/mL of PMA (phorbol 12-myristate 13-acetate) together with 1μM of ionomycin (Sigma Chemical Co, Israel).

### Cytokine secretion measurements

Antigen-specific T-cells were plated onto round 96-well plates in medium containing RPMI-1640 supplemented with 2.5% fetal calf serum (FCS), 100 U/ml penicillin, 100 μg/ml streptomycin, 50μM β-mercaptoethanol, and 2mM L-glutamine. Each of the 96 wells had a final volume of 200μl and contained 10^4^ T-cells, 5x10^5^ irradiated (25 gray) spleen cells, as APC, and 5μg/ml of MOG p35-55. In addition, the relevant peptide was added. Each treatment was made with quadruplicate. Analysis of IFN-γ, IL-4 and TNFα secretion was performed by ELISA 24 hours after cell activation according to standard protocols from R&D systems.

Mouse Femora and tibiae BM cells were collected and cultured in RPMI medium containing FBS (10%), L-glutamine (1%), sodium pyruvate (1%), Pen-strep (1%), and 10 ng/ml recombinant CSF-1 (Peprotech). On day 7, cells were stimulated by either (*i*) LTA, (*ii*) LPS, or (*iii*) PAM3CSK4 (1 μg/ml), in the presence of the HTLV FP at 10μM. Media was collected either 5 hours following activation (for TNF-α detection) or 22 hour following activation (for IL-6 detection) and secretion levels were determined according to standard protocols from R&D systems.

Human peripheral T lymphocytes were isolated from whole blood of healthy donors and were incubated in a complete RPMI growth medium (500 ml RPMI Ca/Mg + heat inactivated FCS (10% final) + 5 ml 200 mM L-Glu (2 mM final) +5 ml 100 M Na pyruvate (1 mM final) + 5 ml Pen Strep antibiotics). Cells were activated using CD3 and CD28 antibodies, in the presence of relevant peptides at 10μM. Media was collected 48 hours following activation and secretion of IL2 and IFN-γ was determined according to standard protocols from R&D systems.

#### RNA isolation and quantitative real time PCR (qRT-PCR)

Antigen-specific T-cells were plated onto round 12-well plates (10^6^ cells/ well) and activated with 5x10^5^ irradiated (25 gray) APC and 5μg/ml of MOG p35-55 in the presence or absence of relevant peptides. Total RNA from cells was isolated 24 hours following activation using the NucleoSpin RNA II kit (Macherey-Nagel, Duren, Germany). 2μg aliquot of the total RNA was reverse transcribed into cDNA using Bio-RT (Bio-Lab, Jerusalem, Israel), dNTPs and random hexamer primers. qRT-PCR was performed on Step One Plus, ABI instrument (Applied Biosystems, Grand Island, NY, USA) using SYBR Green PCR Master Mix (Quanta BioSciences, Gaithersburg, MD, USA). The values for the specific genes were normalized to Rpl13a (mouse) as housekeeping controls and the data are described in arbitrary units. PCR reactions were performed in duplicate. The specific primers used for qRT-PCR are available on request.

### T-bet and Gata3 expression detected by FACS

Antigen-specific T-cells were plated onto round 12-well plates (10^6^ cells/ well) and activated with 5x10^5^ irradiated (25 gray) APC and 5μg/ml of MOG p35-55 in the presence or absence of relevant peptides. Cells were washed with PBS, blocked (5% Donkey serum, 2% BSA and 0.1% Triton in PBS) and fixed with 4% Paraformaldehyde (PFA) 24 hour following activation. Cells were then stained with Gata3-FITC and T-bet-APC fluorochrome-labeled monoclonal mouse antibodies (purchased from Miltenyi Biotec) according to Miltenyi Biotec protocols. Samples were then collected using LSR-II flow cytometer and analyzed with FlowJo cell analysis software.

### Induction of Experimental Autoimmune Encephalomyelitis (EAE)

EAE was induced in 9-week-old wild type and homozygous C57BL/6 female mice (Harlan Laboratories Israel/ Weizmann Institute animal facilities) by injecting a peptide comprising residues 35–55 of mouse myelin oligodendrocyte glycoprotein (MOG35–55; PolyPeptide Laboratories, Strasbourg, France). Mice were injected subcutaneously above the lumbar spinal cord with 100 μl of emulsion containing 200 μg/mouse of the encephalitogenic peptide in complete Freund’s adjuvant (BD-Difco) enriched with 250 μg/mouse of heat-inactivated Mycobacterium tuberculosis (BD-Difco) at 0 days post-induction (DPI). The HTLV FP was dissolved in PBS and added to the emulsion (1mg/kg). Pertussis toxin (Enzo Life Sciences) at a dose of 300 ng per mouse was injected intraperitoneally immediately after the encephalitogenic injection, as well as at 0 DPI. EAE disease was scored using a five-point grading with 0 for no clinical disease; 1, tail weakness; 2, paraparesis (incomplete paralysis of one or two hindlimbs); 3, paraplegia (complete paralysis of one or two hindlimbs); 4, paraplegia with forelimb weakness or paralysis; 5, moribund or dead animals. The mice were examined daily.

### Cytotoxicity assay

Aliquots of 10^4^ cells were distributed onto a 96-well plate in the presence of 1.25–40μM of the relevant peptides for 16 or 72 hours. Following incubation, XTT reaction solution (benzene sulfonic acid hydrate and N-methyl dibenzopyrazine methyl sulfate, mixed in a proportion of 50:1), was added for 2 hours. Optical density was read at 450-nm wavelength. The percentage of toxicity was calculated relative to the control, 10^4^ cells in medium with no peptide added.

### Statistical analysis

Samples sizes were chosen with adequate statistical power on the basis of past experience and literature. Differences between group means were tested using student’s *t*-test when the experiment contained two groups, or one-way ANOVA (followed by a Tukey *post hoc* test) when the experiment contained more than two groups. P< 0.05 was considered significant. Analyses were done using GraphPad Prism (data analysis software) version 6.05. (*P≤0.05, **P≤0.01, ***P≤0.001). Results are displayed as mean ±SEM.

### Ethics statement

All experiments involving animals were conducted under the approval of the IACUC of the Weizmann Institute, permit numbers: 26980516–3 (*in-vitro* T-cell activation assays) and 29650816–3 (Experimental Autoimmune Encephalomyelitis), which were performed in accordance to their relevant guidelines and regulations. The facility where this research was conducted is accredited by AAALAC and has an approved Office of Laboratory Animal Welfare (OLAW) Assurance (#A5005-01). The facility operates according to the guide for the care and use of laboratory animals 8th edition by the national research council. All procedures were conducted by trained personnel under the supervision of veterinarians and all invasive clinical procedures were performed while animals were anesthetized.

Human peripheral T lymphocytes were isolated from whole blood of healthy adult donors that provided written informed consent under the regulations and authorization of the Weizmann Institutional Review Board, Project 247–2.

## Supporting information

S1 Fig Peptides are not toxic to mouse MOG35-55–antigen specific T cells at concentrations used in this study. Viability of cells was analyzed by an XTT cytotoxicity assay. MOG35-55–antigen specific T cells were incubated with peptides at concentrations ranging from 40μM to 2.5μM in serial dilutions (from black to light gray respectively). (*A*) Viability was analyzed following overnight (16 hours) incubation with peptides. (*B*) Viability was analyzed following 72 hour incubation with the peptide. The data is presented as mean percent viability. Error bars represent ± S.E.M. n = 4.
(TIF)

S2 Fig BM-derived macrophages activation is not affected by HTLV FP treatment. Mouse BM-derived macrophages were isolated, grown and stimulated by either (*i*) LTA, (*ii*) LPS, or (*iii*) PAM3CSK4 (1 μg/ml), TLR 2/6, 4/4, and 2/1 ligands, respectively, in the presence of the HTLV FP at 10μM. Media was collected either 5 hours following activation (for TNF-α detection) or 22 hour following activation (for IL-6 detection) and cytokines secretion was measured by ELISA assay. HTLV FP treatment does not affect BM-derived macrophages activation. n = 3. One-way ANOVA was used for statistical analysis. ns, not significant.
(TIF)

S3 Fig Administration of HTLV FP alleviates MOG35-55-induced EAE. EAE was induced in C57BL/6 female mice that were either treated with a single dose of HTLV FP or vehicle. Two indexes to measure clinical disease severity are displayed. (*A*) Direct clinical measurement of EAE phenotype in a 5-point scale with increased disease symptoms correlating with higher score value. The data is presented as mean EAE clinical score. (*B*) Mice were weighed the day before EAE induction, and the change (as a percentage) in weight was recorded. The data is presented as mean change from the initial weight. Student’s *t-*test was used for statistical analysis. *P<0.05; **P<0.01;***P<0.001.
(TIF)

S4 Fig Peptides are not toxic to primary human T cells at concentrations used in this study. Viability of cells was analyzed by an XTT cytotoxicity assay. MOG35-55–antigen specific T cells were incubated with peptides at concentrations ranging from 40μM to 2.5μM in serial dilutions (from black to light gray respectively). Viability was analyzed following overnight (16 hours) incubation with peptides. The data is presented as mean percent viability. Error bars represent ± S.E.M. n = 4.
(TIF)

S5 Fig Monitoring of T-bet expression in activated and non-activated T-cells. MOG_35-55_–antigen specific T-cells were activated by irradiated MOG_35-55_ presenting APCs. Samples were fixed in 4% PFA and stained with T-bet-APC antibody 24, 48 and 72 hours following activation. Analysis was performed using LSR-II flow cytometer and FlowJo cell analysis software. (*A*) Gating on lymphocytes. (*B*) Gating on T-bet positively stained cells. (*C*) T-bet expression in Non-activated and activated T-cells. (*D*) T-bet expression in Non-activated and activated T-cells over the course of 72 hours. An increase in T-bet expression is observed upon T-cell activation. n = 3.
(TIF)

S6 Fig Changes in T-bet expression in activated T-cells over 72 hours. MOG_35-55_–antigen specific T-cells were activated by irradiated MOG_35-55_ presenting APCs. Samples were fixed in 4% PFA and stained with T-bet-APC antibody 24, 48 and 72 hours following activation. Analysis was performed using LSR-II flow cytometer and FlowJo cell analysis software. T-bet expression decreases 72 hour following activation compared to its expression 24 and 48 hours post activation. n = 4. One-way ANOVA was used for statistical analysis. *P<0.05.
(TIF)

S7 Fig Monitoring of Gata3 expression in activated and non-activated T-cells. MOG_35-55_–antigen specific T-cells were activated by irradiated MOG_35-55_ presenting APCs. Samples were fixed in 4% PFA and stained with T-bet-APC antibody 24, 48 and 72 hours following activation. Analysis was performed using LSR-II flow cytometer and FlowJo cell analysis software. (*A*) Gating on lymphocytes. (*B*) Gating on Gata3 positively stained cells. (*C*) An example of an increase in Gata3 expression. (*D*) Gata3 expression in Non-activated and activated T-cells over the course of 72 hours. Gata3 expression is not changed upon T-cell activation. n = 3.
(TIF)

S8 Fig NPS secondary structure prediction analysis of the HTLV FP sequence. Secondary structure was predicted based on the PHD [109], DSC [110] and MLRC [111] methods, and a secondary structure consensus was generated.
(TIF)

S1 Movie Vehicle treated mouse exhibiting a clinical score of 3 with complete hind limb paralysis at DPI 9. Mouse is seen roaming in the cage by dragging itself using his forelimbs only as a result of paralysis.
(MP4)

S2 Movie A cage of vehicle treated mice with 4 out of 5 mice exhibiting a clinical score of 3 at DPI 9. Mice are seen still at the back of the cage as a result of hind limb paralysis.
(MP4)

S3 Movie A cage of HTLV FP treated mice with no observed clinical symptoms at DPI 9. All mice seem healthy and are seen roaming freely in the cage.
(MP4)

S4 Movie A comparison of cages with vehicle treated mice (right) and with HTLV FP treated mice (left) as observed at DPI 14. Only 1 out of 5 mice that were treated with HTLV FP exhibits mild clinical symptoms while the rest seem completely healthy with no observed clinical signs. In contrast, all vehicle treated mice exhibit severe clinical symptoms.
(MP4)

S5 Movie A cage of HTLV Scr treated mice exhibiting clinical EAE symptoms at DPI 17. Mice are seen still at the back of the cage as a result of partial hind limb paralysis.
(MP4)

S6 Movie A cage of HTLV FP treated mice with mild clinical symptoms at DPI 17. Mice are seen roaming freely in the cage with 3 out of 5 mice exhibiting no clinical manifestation.
(MP4)
