# Supplementary figures and images for: The HTLV-1 gp21 fusion peptide inhibits antigen specific T-cell activation in-vitro and in mice
Source: PLoS Pathog. 2018 May 4;14(5):e1007044. doi: 10.1371/journal.ppat.1007044 (PMC5955599; doi:10.1371/journal.ppat.1007044)

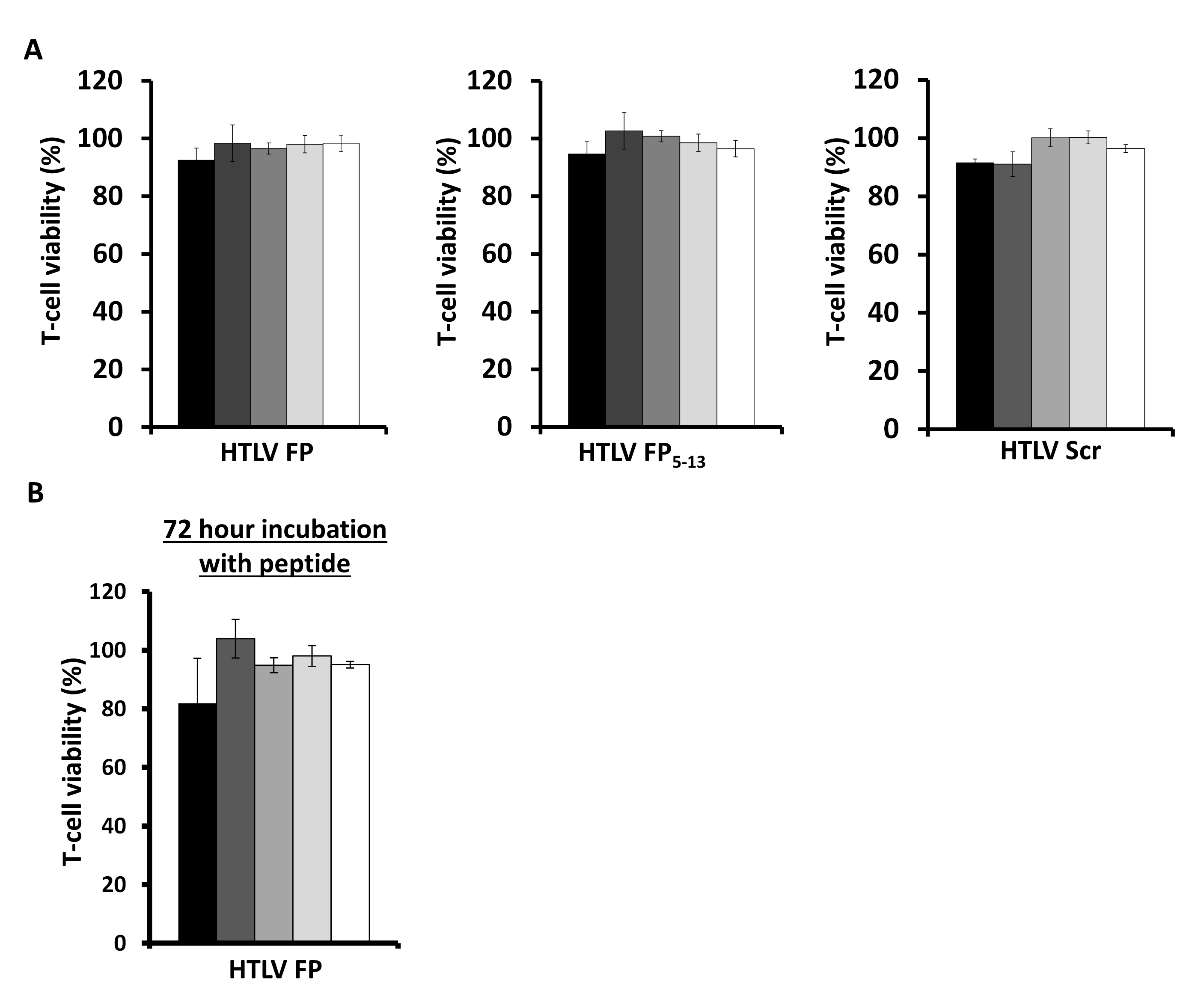

Supplement: S1 Fig — Viability of cells was analyzed by an XTT cytotoxicity assay. MOG35-55–antigen specific T cells were incubated with peptides at concentrations ranging from 40μM to 2.5μM in serial dilutions (from black to light gray respectively). (A) Viability was analyzed following overnight (16 hours) incubation with peptides. (B) Viability was analyzed following 72 hour incubation with the peptide. The data is presented as mean percent viability. Error bars represent ± S.E.M. n = 4. (TIF) [file ppat.1007044.s001.tif]

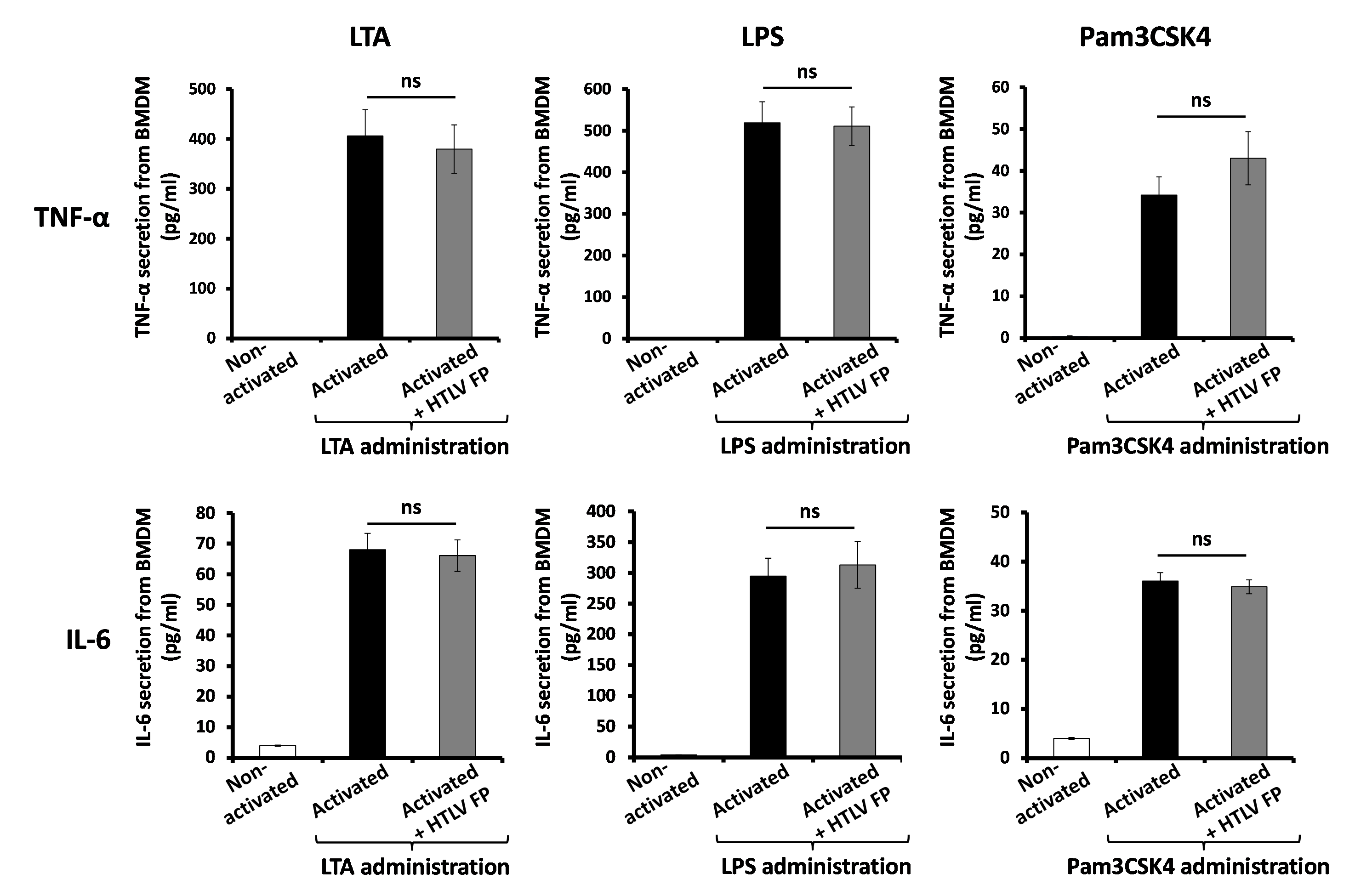

Supplement: S2 Fig — Mouse BM-derived macrophages were isolated, grown and stimulated by either (i) LTA, (ii) LPS, or (iii) PAM3CSK4 (1 μg/ml), TLR 2/6, 4/4, and 2/1 ligands, respectively, in the presence of the HTLV FP at 10μM. Media was collected either 5 hours following activation (for TNF-α detection) or 22 hour following activation (for IL-6 detection) and cytokines secretion was measured by ELISA assay. HTLV FP treatment does not affect BM-derived macrophages activation. n = 3. One-way ANOVA was used for statistical analysis. ns, not significant. (TIF) [file ppat.1007044.s002.tif]

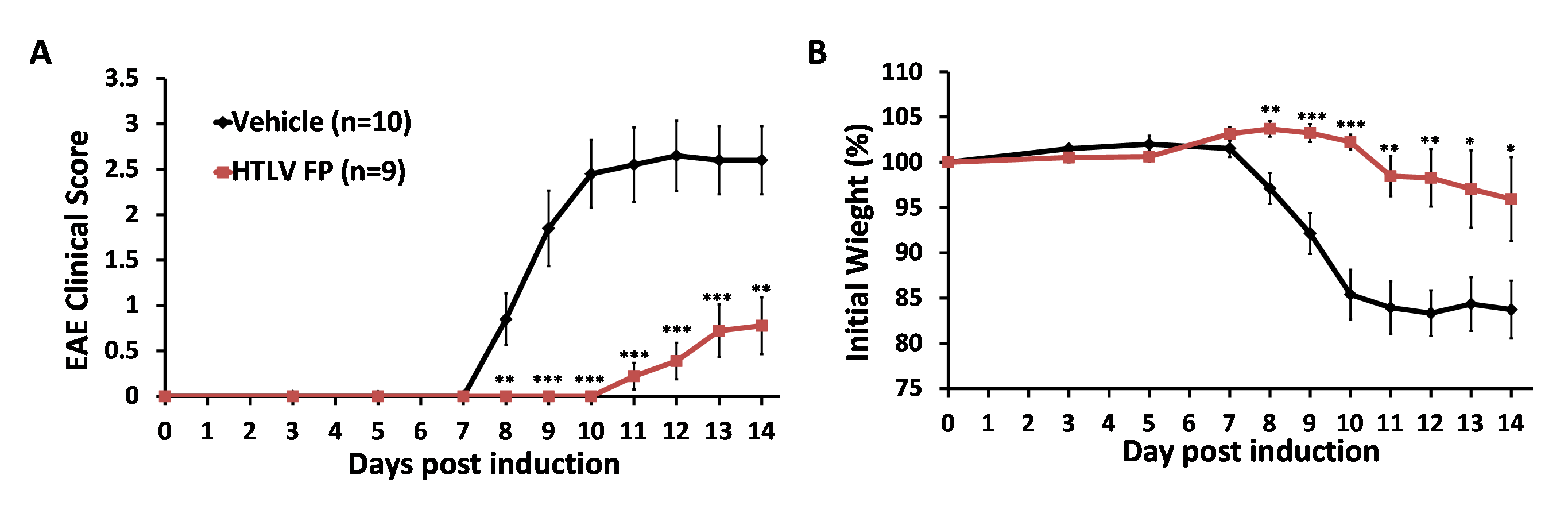

Supplement: S3 Fig — EAE was induced in C57BL/6 female mice that were either treated with a single dose of HTLV FP or vehicle. Two indexes to measure clinical disease severity are displayed. (A) Direct clinical measurement of EAE phenotype in a 5-point scale with increased disease symptoms correlating with higher score value. The data is presented as mean EAE clinical score. (B) Mice were weighed the day before EAE induction, and the change (as a percentage) in weight was recorded. The data is presented as mean change from the initial weight. Student’s t-test was used for statistical analysis. *P<0.05; **P<0.01;***P<0.001. (TIF) [file ppat.1007044.s003.tif]

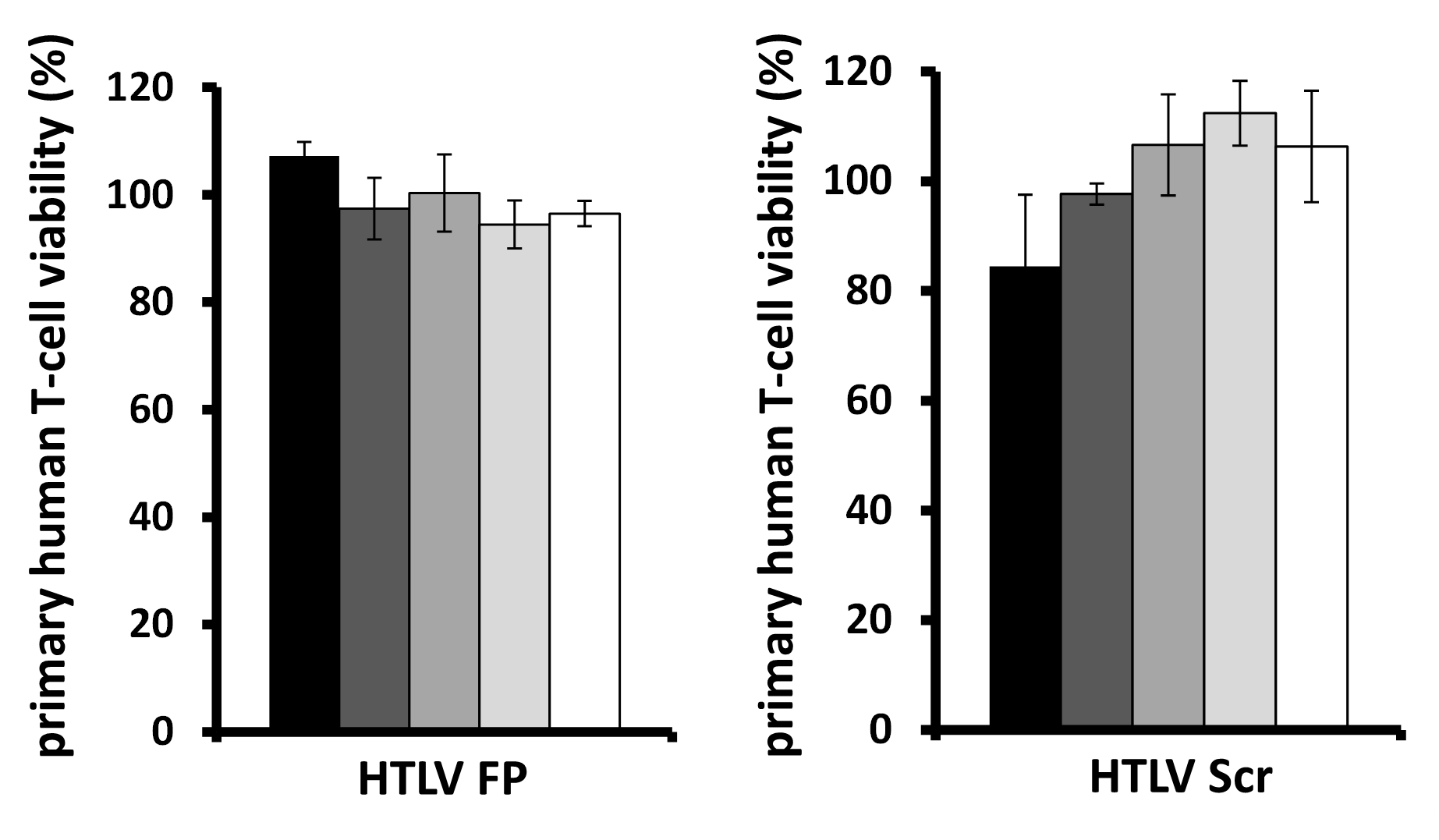

Supplement: S4 Fig — Viability of cells was analyzed by an XTT cytotoxicity assay. MOG35-55–antigen specific T cells were incubated with peptides at concentrations ranging from 40μM to 2.5μM in serial dilutions (from black to light gray respectively). Viability was analyzed following overnight (16 hours) incubation with peptides. The data is presented as mean percent viability. Error bars represent ± S.E.M. n = 4. (TIF) [file ppat.1007044.s004.tif]

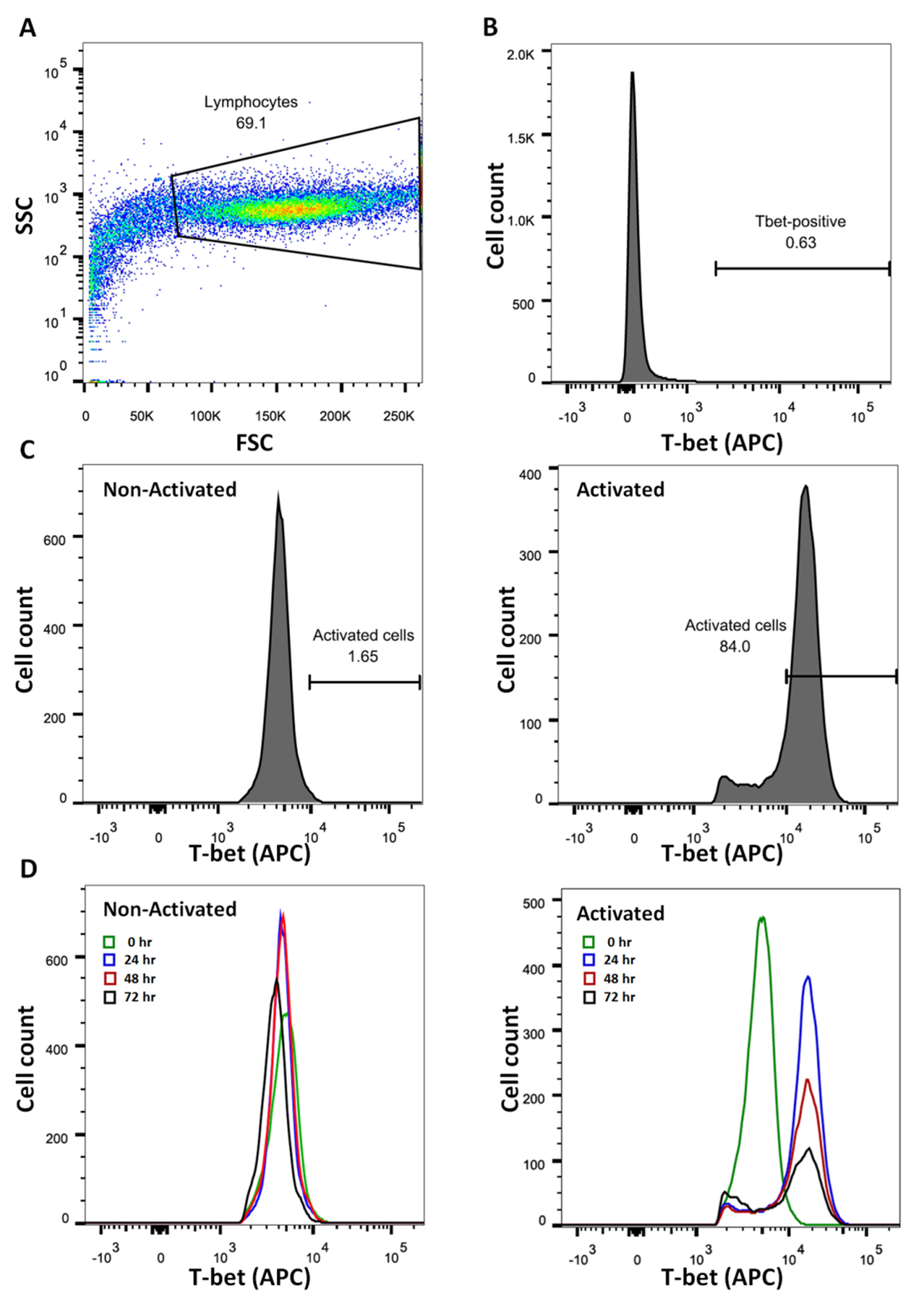

Supplement: S5 Fig — MOG35-55–antigen specific T-cells were activated by irradiated MOG35-55 presenting APCs. Samples were fixed in 4% PFA and stained with T-bet-APC antibody 24, 48 and 72 hours following activation. Analysis was performed using LSR-II flow cytometer and FlowJo cell analysis software. (A) Gating on lymphocytes. (B) Gating on T-bet positively stained cells. (C) T-bet expression in Non-activated and activated T-cells. (D) T-bet expression in Non-activated and activated T-cells over the course of 72 hours. An increase in T-bet expression is observed upon T-cell activation. n = 3. (TIF) [file ppat.1007044.s005.tif]

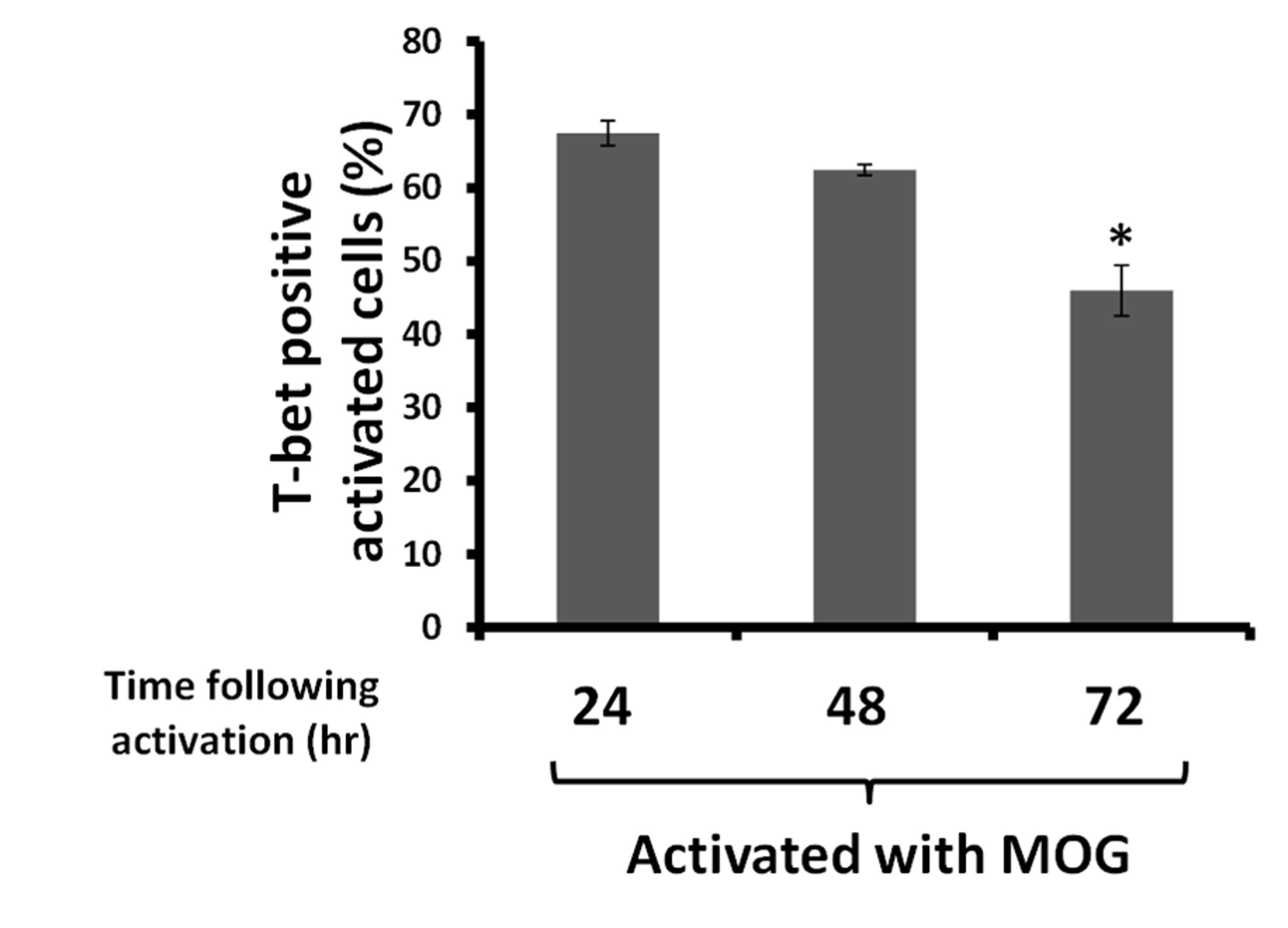

Supplement: S6 Fig — MOG35-55–antigen specific T-cells were activated by irradiated MOG35-55 presenting APCs. Samples were fixed in 4% PFA and stained with T-bet-APC antibody 24, 48 and 72 hours following activation. Analysis was performed using LSR-II flow cytometer and FlowJo cell analysis software. T-bet expression decreases 72 hour following activation compared to its expression 24 and 48 hours post activation. n = 4. One-way ANOVA was used for statistical analysis. *P<0.05. (TIF) [file ppat.1007044.s006.tif]

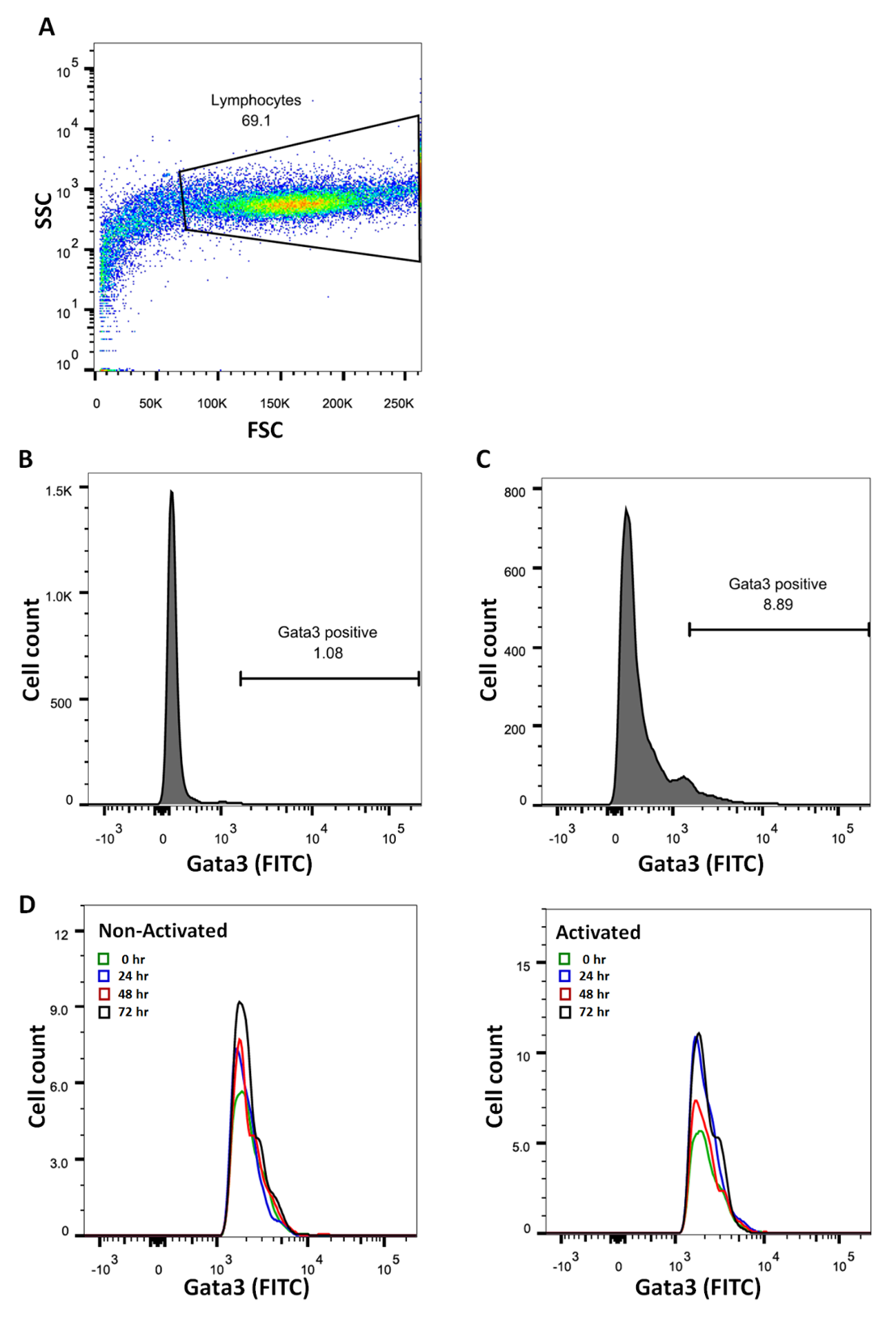

Supplement: S7 Fig — MOG35-55–antigen specific T-cells were activated by irradiated MOG35-55 presenting APCs. Samples were fixed in 4% PFA and stained with T-bet-APC antibody 24, 48 and 72 hours following activation. Analysis was performed using LSR-II flow cytometer and FlowJo cell analysis software. (A) Gating on lymphocytes. (B) Gating on Gata3 positively stained cells. (C) An example of an increase in Gata3 expression. (D) Gata3 expression in Non-activated and activated T-cells over the course of 72 hours. Gata3 expression is not changed upon T-cell activation. n = 3. (TIF) [file ppat.1007044.s007.tif]

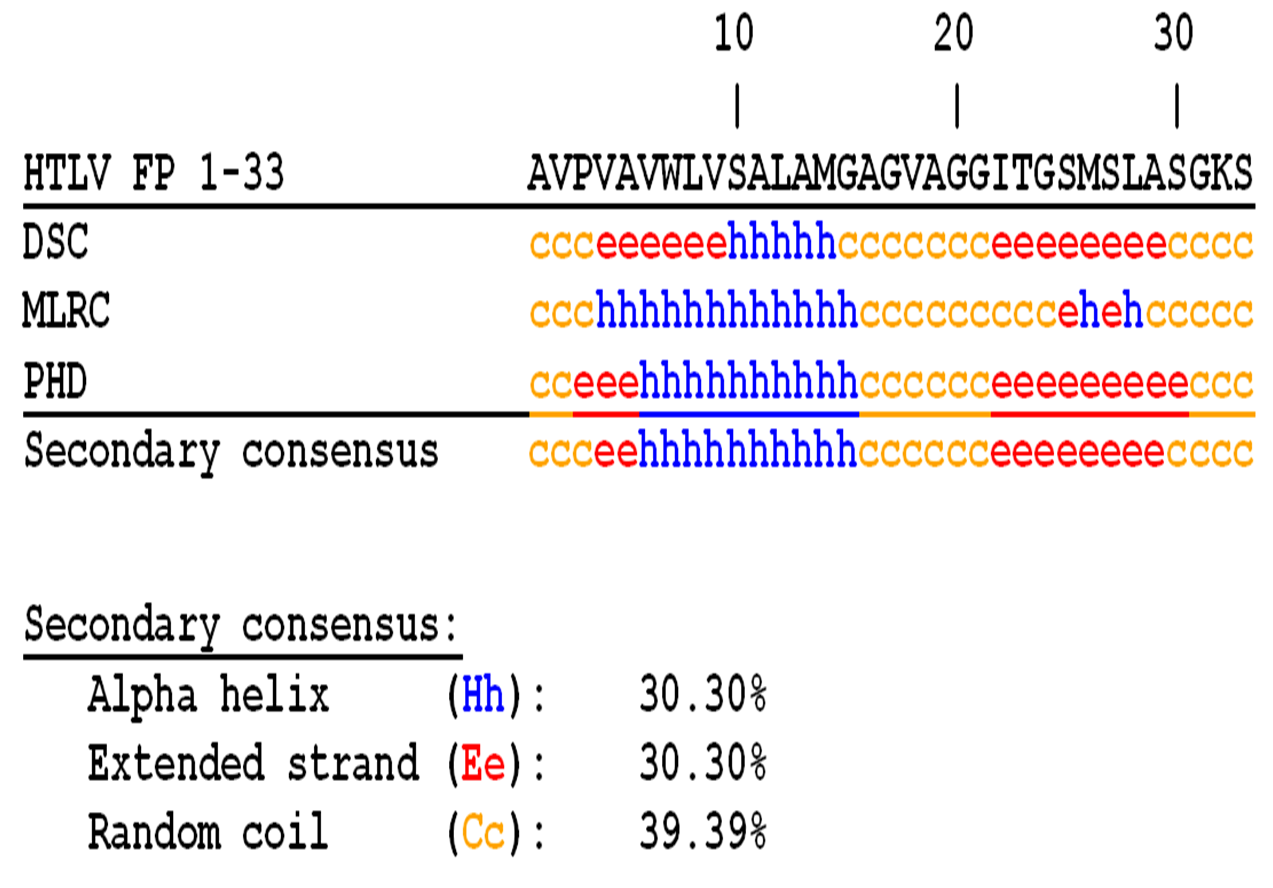

Supplement: S8 Fig — Secondary structure was predicted based on the PHD [109], DSC [110] and MLRC [111] methods, and a secondary structure consensus was generated. (TIF) [file ppat.1007044.s008.tif]
